# Hypoxia increases the activity of oncolytic adenoviruses through HIF-2α-stimulated E1A expression

**DOI:** 10.1038/s41392-026-02895-y

**Published:** 2026-08-11

**Authors:** Egon J. Jacobus, Véronique N. Lafleur, Kerry D. Fisher, David R. Mole, Leonard W. Seymour

**Affiliations:** 1 Department of Oncology, University of Oxford, Oxford, UK; 2 Nuffield Department of Medicine Research Building, University of Oxford, Oxford, UK

**Keywords:** Nanobiotechnology, Cancer therapy, Cancer microenvironment, Microbiology

## Abstract

Hypoxia, a hallmark of solid tumors, poses a significant challenge in cancer therapy due to its association with poor prognosis and resistance to conventional treatments. Oncolytic viruses represent a promising treatment strategy, as they selectively replicate within cancer cells and lyse them, potentially including those in hypoxic tumor regions. Here, we examined how hypoxic conditions influence the activity of enadenotucirev (EnAd), a clinically relevant group B oncolytic adenovirus previously detected in hypoxic areas of xenograft tumors. We demonstrated that hypoxia enhances virus production by boosting transcription and translation of immediate-early, early, and late adenoviral genes. The immediate-early gene *E1A* was upregulated within 2 h (17-fold) after virus entry under hypoxia, driven by a conserved hypoxia-response element (HRE) in its promoter. Mechanistic studies revealed that the hypoxia-inducible factor (HIF)-2α and HIF-1β heterodimers bind to this HRE, transactivating *E1A*. By inducing E1A expression, hypoxia also elevated viral genome synthesis, structural protein production, and therapeutic transgene expression, underscoring the potential of EnAd to target the hypoxic tumor microenvironment. This is the first report of a functional HRE in a human adenovirus, conserved across 59 adenovirus genotypes, and identifies hypoxia as a driver of enhanced oncolytic activity with implications for adenovirus-based therapies in solid tumors.

## Introduction

Hypoxia is a defining feature of most solid tumors and can shape stromal and immune cell behavior in ways that promote tumor progression.^1^ Increased hypoxic burden is associated with poorer clinical outcomes and resistance to radio-, chemo-, and immunotherapy.^2–4^ Thus, hypoxic tumor regions remain an important therapeutic challenge. Oncolytic viruses could target the hypoxic fraction of tumors owing to their selective replication, induction of cell lysis, and cell-to-cell spread, thereby reaching hypoxic areas that may otherwise be inaccessible to other therapeutic platforms due to poor tumor perfusion.

Oncolytic viruses have shown promising clinical outcomes,^5^ leading to the approval of Imlygic® for unresectable melanoma in the US and Europe,^6^ and more recently, the licensing of a similar virus in Japan for glioblastoma treatment.^7^ Enadenotucirev (EnAd, formerly ColoAd1) is a chimeric Ad11p/Ad3 adenovirus engineered through directed evolution to selectively target and robustly replicate in epithelial cancer cells.^8^ It was prioritized for intravenous delivery due to its stability in blood and low prevalence of neutralizing antibodies. While early clinical trials demonstrated safe intravenous delivery and infection of remote tumor deposits,^9,10^ relatively little is known about how this adenovirus performs under hypoxic conditions.

When viruses are administered intravenously, the first tumor cells encountered are likely to be well-oxygenated. As the virus spreads, or through perfusion changes within the vascular bed, it may subsequently encounter hypoxic regions with partial oxygen tensions (pO_2_) below 2%.^11^ We have previously found EnAd infection foci within hypoxic areas of tumor xenografts.^12^ Moreover, different classes of oncolytic viruses vary in their ability to target hypoxic cancer cells in vitro.^13–16^

Hypoxia poses a set of challenges that can limit viral replication, including reduced global transcription and translation, altered metabolism, and altered antiviral signaling. Many of these changes are governed by transcriptional adaptation through the oxygen-sensitive stabilization of hypoxia-inducible factor subunits (HIF)-1α and HIF-2α.^17^ Under normoxia, prolyl-hydroxylase domain proteins (PHDs) hydroxylate the α-subunits, which are recognized by the E3 ubiquitin ligase VHL (Von–Hippel–Lindau tumor suppressor) and consequently targeted for degradation.^18^ In hypoxia, PHDs are inactive, leading to stabilization of HIF-1α and HIF-2α. These heterodimerize with the constitutively expressed HIF-1β subunit and translocate into the nucleus.^19^ The active heterodimer binds to the R/CGTG consensus sequence, known as the hypoxia-response element (HRE), within the promoters of hypoxia-responsive genes, thereby leading to transcriptional activation.^20^

Herein, we set out to determine which stages of the oncolytic adenovirus infection cycle are influenced by hypoxia. Following assessment of EnAd distribution across oxygen gradients in tumor xenografts, we used an in vitro single-step infection model to evaluate EnAd uptake, entry, replication kinetics, and particle production under normoxic (21% pO_2_) and hypoxic (1% pO_2_) conditions. We further examined the impact of hypoxia on viral transcription and translation, with particular emphasis on immediate-early gene expression. Our findings reveal a positive role for hypoxia in viral production and highlight the supportive role of the HIF pathway in viral immediate-early transcription, offering insights into adenovirus infection in hypoxic tumor regions.

## Results

### EnAd entry, infection kinetics, and virion production in hypoxic cells

**Fig. 1:**
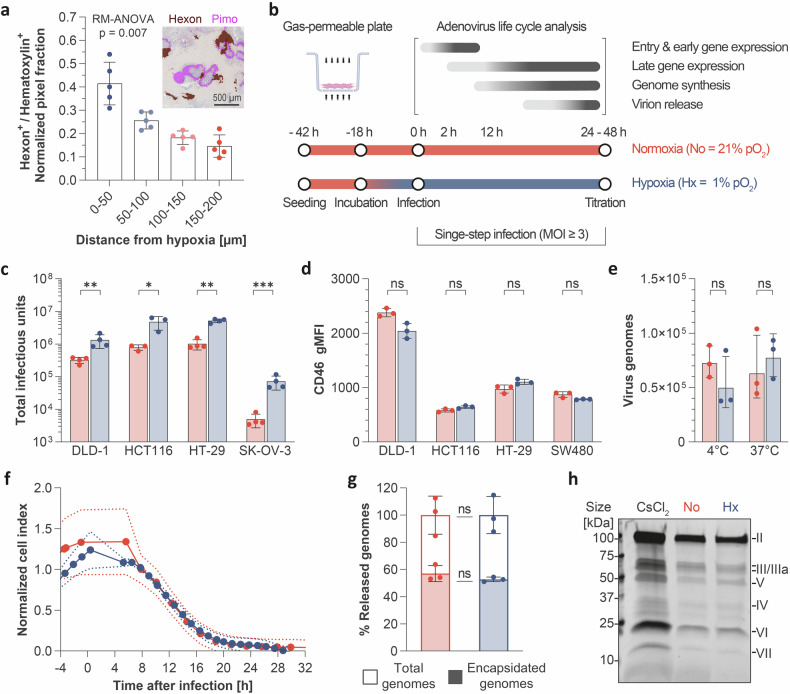
In vivo EnAd infection is prevalent in hypoxic regions, and in vitro hypoxia increases infectious virus particle production without altering entry, genome packaging, infection kinetics, or progeny virion integrity. **a** The spatial proximity of virus foci (hexon^+^) to the cores of hypoxic (pimonidazole^+^, pimo) and non-necrotic (hematoxylin^+^) areas was measured in DLD-1 xenograft tumors 16 days after intravenous administration of enadenotucirev encoding luciferase (EnAd-SA-fLuc). Data are shown in 50-µm bins and assessed by repeated-measures (RM) ANOVA, *n* = 5. Extended data are available in Supplementary Fig. 1. **b** Cancer cell lines were pre-exposed for 18 h to hypoxia (1% pO_2_, Hx, blue) or normoxia (21% pO_2_, No, red), then infected with EnAd-SA-fLuc. The adenovirus life cycle was analyzed at a multiplicity of infection of 3 (MOI), using gas-permeable plates to maintain a constant oxygen tension during infection. **c** Released infectious particles into the supernatant were quantified 30 h post-infection (hpi), *n* = 4; except for HCT116 cells, *n* = 3. **d** CD46 surface expression was measured before infection; geometric mean fluorescence intensity (gMFI) is shown, *n* = 3. **e** In DLD-1 cells, particle attachment during virus entry (2 hpi) was quantified by measuring viral genomes after infection at 4 °C and internalization at 37 °C, *n* = 3. **f** Virus-induced cytotoxicity was tracked via real-time impedance measurement, mean cell index of 4 technical replicates. **g** Genome encapsidation at 30 hpi was assessed by comparing total released viral genomes (untreated, hollow bar) with encapsidated virus genomes (benzonase-treated, filled bar, *n* = 3). **h** Progeny virion integrity was assessed by denaturing electrophoresis of purified virus from supernatants and a CsCl_2_-gradient-purified control; II (hexon), III (penton), IIIa (penton base), V (minor core protein), IV (fiber), VI (core), VII (hexon minor polypeptide). Data are shown as mean ± SD, *P* value: not significant (ns) *P* > 0.05, *P ≤ 0.05, **P ≤ 0.01, ***P ≤ 0.001

Following intravenous EnAd administration to mice bearing DLD-1 tumor xenografts, spatial histological analysis at steady-state infection revealed enrichment of the viral capsid protein hexon within hypoxic and adjacent regions (Fig. 1a and Supplementary Fig. 1). The area fraction with the highest positivity for hexon staining was observed in the first 50 µm band surrounding the core of hypoxic regions, as defined by the center of pimonidazole adduct staining (≤1.3%^21,22^), followed by the 50–100 µm band. Hexon staining was markedly reduced beyond 100 µm, an area predicted to lie within the limits of oxygen diffusion, and therefore well-oxygenated.^22^ Based on these observations, we set out to investigate the influence of hypoxia on EnAd infection in vitro.

To this end, we modeled tumor hypoxia by pre-exposing cancer cells to either a normoxic (21% pO_2_) or a hypoxic (1% pO_2_) environment for 18 h. Importantly, experiments were performed using gas-permeable plates to ensure that cells experience the set oxygen tension over time. We focused on single-step EnAd infections at a high multiplicity of infection (MOI ≥ 3), thereby ensuring that most cells were directly infected (Fig. 1b). Infectious units were measured using a high-dynamic-range titration assay that correlates plaque-forming units with luciferase signal from a reporter EnAd virus expressing firefly luciferase, linked to the major late transcription unit via a splice acceptor site (EnAd-SA-fLuc,^23^ Supplementary Fig. 2). After one infection cycle under hypoxic conditions, the yield of infectious virus particles increased 4- to 14-fold relative to normoxia in human colorectal (DLD-1, HCT116, and HT-29) and ovarian carcinoma cells (SK-OV-3, Fig. 1c). This boost in infectious virus production could result from improvements at any step of the viral life cycle, including uptake and entry, transcription and translation of viral genes, packaging efficiency of virus progeny, or a shorter viral life cycle. We therefore set out to systematically assess how hypoxia affects these steps and the overall duration of the viral life cycle.

Virus particle uptake could depend on the abundance of the high-affinity entry receptor CD46. Hypoxia did not alter the surface expression of CD46 (Fig. 1d). We also demonstrated that hypoxia did not affect the number of surface-bound or internalized virus genomes during incubation at either 4 °C or 37 °C (Fig. 1e). Therefore, we concluded that virus entry remains unchanged in hypoxic cells.

As the duration of the infection cycle could affect viral burst size, we tracked cell detachment caused by viral cytotoxicity (cytopathic effect) using real-time electrical impedance measurements (xCELLigence). Hypoxia did not alter the rate of decrease in impedance (cell index) during EnAd infection, indicating that it did not affect viral life cycle kinetics in DLD-1 cells (Fig. 1f).

To assess the influence of hypoxia on the efficiency of packaging viral genomes into progeny virions, supernatants from infected DLD-1 cells were treated with benzonase to degrade unprotected DNA and to determine the proportion of viral genomes contained within intact capsids. Genome encapsidation rates were similar under normoxia and hypoxia (Fig. 1g). Progeny virions from hypoxic and normoxic DLD-1 cells also showed no difference in protein composition when analyzed by denaturing polyacrylamide gel electrophoresis (Fig. 1h). Thus, hypoxia treatment did not disturb progeny particle assembly or particle integrity.

### Viral immediate-early, early, late, and transgene expression under hypoxia

**Fig. 2:**
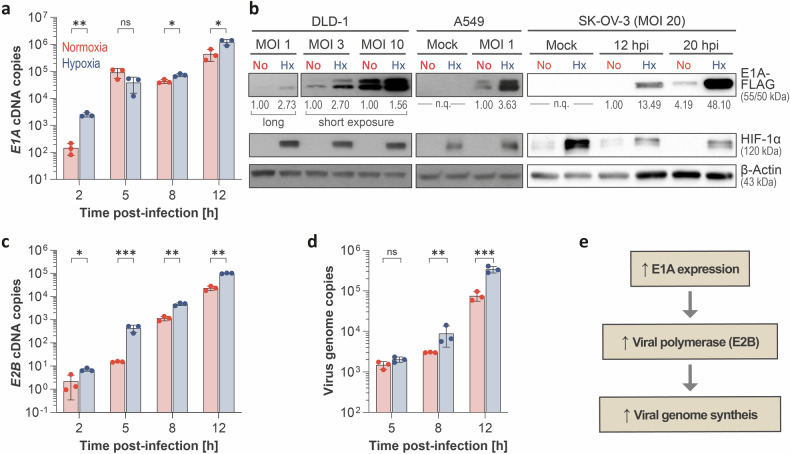
Hypoxia increases immediate-early and early gene expression, and EnAd virus genome production. Cancer cells pre-exposed to hypoxia (Hx) or normoxia (No) were infected with enadenotucirev (EnAd-SA-fLuc) under constant oxygen tension. **a**, **c** *E1A* and *E2B* mRNA copy numbers were measured by qRT-PCR in DLD-1 cells at a multiplicity of infection of 3 (MOI), *n* = 3. **b** In DLD-1, A549, and SK-OV-3 cells infected with EnAd encoding a FLAG-tag at the c-terminus of E1A (EnAd-E1A-cFLAG), E1A protein levels were assessed 8 h post-infection by immunoblotting for FLAG-tagged E1A, HIF-1α (hypoxia incubation control), and β-Actin. Long- and short-exposure images for DLD-1 stem from the same blot. **d** EnAd genome replication in DLD-1 cells was measured by qPCR; MOI = 3, *n* = 3. **e** Through positive feedback, E1A upregulation induces the downstream viral polymerase E2B, promoting genome replication. Data are shown as mean ± SD, *P* value: not significant (ns) *P* > 0.05, *P ≤ 0.05, **P ≤ 0.01, ***P ≤ 0.001. Negative control bands were not quantified (n.q.)

We sought to understand the enhanced viral yield observed in hypoxic conditions by assessing transcript and protein levels of immediate-early adenovirus genes in DLD-1 cells. Hypoxia induced a rapid upregulation of *E1A* transcription, with copy numbers rising to 17-fold above normoxic levels within 2 h (Fig. 2a). *E1A* mRNA levels rose rapidly over time and remained higher in hypoxic conditions at 8 and 12 h post-infection (hpi). Direct measurement of E1A protein was not possible due to the lack of specific antibodies against group B adenovirus E1A. To overcome this, we engineered a recombinant virus encoding a FLAG-tag fused to the carboxy-terminus of *E1A* (EnAd-E1A-cFLAG). This resulted in the detection of two large protein isoforms of E1A (50 and 55 kDa; Supplementary Figs. 3 and 4). Consistent with the mRNA findings, hypoxia-specific increases in both E1A protein isoforms were observed in DLD-1 and A549 cells at 8 hpi. A similar effect was seen in the less permissive cell line SK-OV-3 at an MOI of 20 (Fig. 2b). Thus, hypoxia markedly increased expression of E1A, the earliest adenoviral gene transcribed upon infection.

The *E2B* gene encodes the adenoviral polymerase and is induced after E1A expression.^24^ *E2B* mRNA levels were higher under hypoxia than under normoxia and remained elevated over 12 h, reaching up to 30-fold more mRNA copies under hypoxia at 5 hpi (Fig. 2c). As with E1A, E2B protein levels could not be measured directly due to a lack of suitable antibodies. However, using E2B polymerase activity as a proxy for its abundance, we found that increased *E2B* transcription in hypoxic cells translated to a 4.5-fold increase in EnAd genome synthesis at 12 hpi (Fig. 2d, e). Hypoxia similarly enhanced E1A expression and genome synthesis in the group C adenovirus serotype 5 (Ad5), which is widely employed for gene therapy and oncolytic virotherapy (Supplementary Fig. 5).

**Fig. 3:**
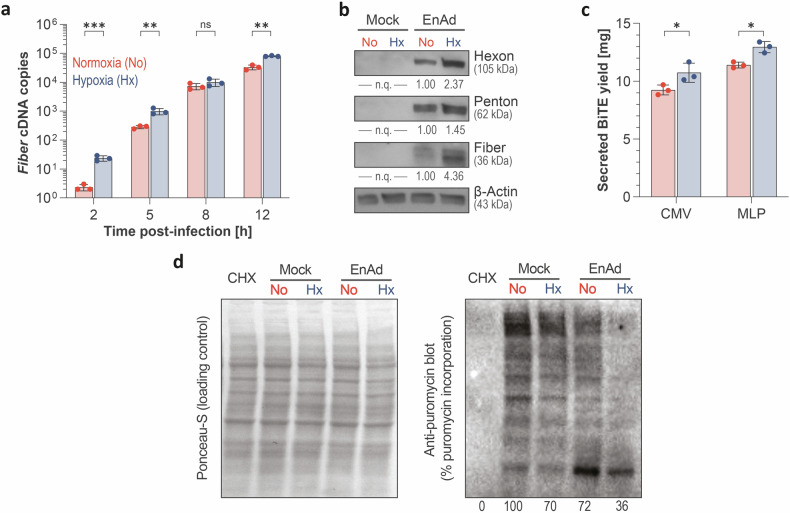
Hypoxia enhances the expression of late virus genes and virus-delivered transgenes. DLD-1 cells pre-exposed to hypoxia (Hx) or normoxia (No) were infected with enadenotucirev (EnAd-SA-fLuc) at a multiplicity of infection of 3 (MOI) under constant oxygen tension. **a** *Fiber* mRNA copies were measured by qRT-PCR, *n* = 3. **b** Late viral proteins (hexon, penton, fiber) were detected by immunoblotting at 24 h post-infection (hpi). **c** Therapeutic transgene expression was assessed in cells infected with EnAd encoding a bispecific T cell engager (BiTE) driven by an independent cytomegalovirus (CMV) promoter or coupled to the adenovirus major late promoter (MLP). BiTE secretion was quantified by dot-blot and densitometry analysis, *n* = 3. **d** Global protein translation was assessed by puromycin incorporation into nascent polypeptides at 24 hpi. Translational inhibition with cycloheximide (CHX) served as a negative control. Blots show total protein after denaturing electrophoresis, Ponceau-S staining, and immunoblotting with anti-puromycin antibodies. Data are shown as mean ± SD, *P* value: not significant (ns) *P* > 0.05, *P ≤ 0.05: **P ≤ 0.01, ***P ≤ 0.001. Negative control bands were not quantified (n.q.)

We then investigated whether hypoxia affected late viral gene and transgene expression. *Fiber* mRNA, a late viral gene under the major late promoter (MLP), was upregulated in hypoxia as early as 2 hpi and reached up to fourfold higher levels than in normoxia at 12 hpi (Fig. 3a). Consistently, the translation of the three main capsid proteins, fiber, hexon, and penton, was also increased in hypoxia (Fig. 3b). In addition, the secretion of an EnAd-encoded therapeutic transgene, such as the EpCAM/CD3 bispecific T-cell engager (BiTE),^25^ rose by 15% under hypoxia compared with normoxia (Fig. 3c and Supplementary Fig. 6). This increase was smaller than that observed in viral genes and was similar whether BiTE expression was driven by a cytomegalovirus (CMV) promoter or the MLP.

To understand the extent to which oxygen tension and/or viral infection affect translation, we measured global protein production by detecting puromycin incorporation into nascent polypeptide chains (Fig. 3d). Hypoxia alone led to a partial shutdown of translation, as evidenced by a 30% reduction. Virus infection under normoxia led to a similar reduction, whereas the combination of hypoxia and infection further suppressed global protein translation in an additive manner. Despite significant inhibition of the host’s translation machinery under hypoxia, EnAd enhanced viral transcription and translation of immediate-early, early, and late viral genes and transgenes.

### Induction of E1A expression in hypoxic cultures

**Fig. 4:**
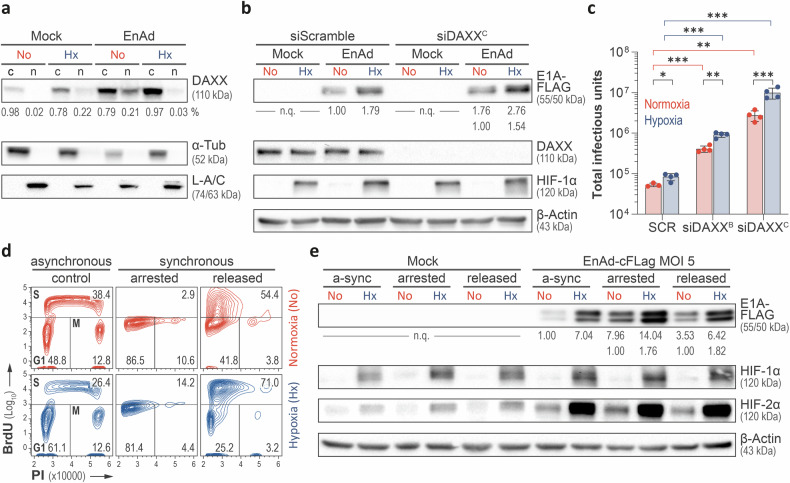
DAXX silencing and G1 arrest facilitate E1A expression in both normoxia and hypoxia. DLD-1 cells pre-exposed to hypoxia (Hx) or normoxia (No) were infected with enadenotucirev (EnAd-E1A-cFLAG or EnAd-SA-fLuc) at a multiplicity of infection of 3 (MOI) under constant oxygen tension. **a** Cytoplasmic (c) and nuclear (n) levels of the Death domain-associated protein (DAXX) were analyzed 8 h post-infection (hpi); nuclear loading control was α-Tubulin (α-Tub), and cytoplasmic LaminA/C (L-A/C). **b**, **c** Cells were transfected with siRNA B or C targeting DAXX before hypoxia exposure and infection. FLAG-tagged E1A, DAXX, HIF-1α and β-Actin were detected by immunoblot at 8 hpi, and virus production was measured at 30 hpi, *n* = 4. **d** Cell-cycle-synchronous (obtained by double thymidine block/arrest), block-released, and asynchronous cultures were tracked by flow cytometry detection of bromodeoxyuridine (BrdU) and propidium iodide (PI) incorporation before infection. The percentages of cells in G1, M, or S-Phase from a representative sample are shown. **e** The cell cycle dependence of E1A upregulation was assessed in the same populations at 10 hpi by immunoblotting for FLAG-tagged E1A, HIF-1α, HIF-2α and β-Actin. Data are shown as mean ± SD, *P* value: not significant (ns) *P* > 0.05, *P ≤ 0.05, **P ≤ 0.01, ***P ≤ 0.001. Negative control bands were not quantified (n.q.)

We explored mechanisms underlying hypoxia-induced E1A upregulation, including those involved in *E1A* locus activation and in the cell cycle. Death domain-associated protein (DAXX) is a cellular transcriptional repressor, key to adenoviral immediate-early transcription.^26^ The repressive activity of DAXX relies on its nuclear localization. Therefore, we assessed whether hypoxia alters DAXX cellular localization, thereby promoting E1A expression. In mock-infected cells, DAXX was predominantly cytoplasmic at both oxygen tensions (Fig. 4a). EnAd infection in normoxic cells increased the nuclear DAXX fraction compared with mock controls. Conversely, in hypoxic infected cells, DAXX remained almost exclusively cytoplasmic, coinciding with the described hypoxia-driven E1A upregulation. Thus, we hypothesized that DAXX depletion induces E1A expression in normoxia to levels similar to those induced by hypoxia, with a concomitant minimal effect on E1A levels in hypoxic infected cells. However, siRNA-mediated depletion of DAXX led to increased E1A expression under both normoxic and hypoxic conditions (Fig. 4b). In addition, DAXX knockdown boosted virus titers by up to 52-fold in normoxia and 118-fold in hypoxia at 30 hpi compared with scrambled siRNA controls (Fig. 4c). Yet, virus production under normoxia remained lower than in hypoxic cells, even in knockdown conditions. Therefore, the specific upregulation of E1A under hypoxia appeared independent of DAXX upregulation and hypoxia-triggered nuclear localization, whereas DAXX depletion boosted E1A and virus production.

Since hypoxia causes cells to accumulate in the G1 phase of the cell cycle,^27^ we explored whether this state favored E1A expression in cell-cycle synchronization experiments using a double thymidine block. While asynchronous cells spread across G1, S, and M phases, synchronized cells were arrested in G1 phase under both normoxic and hypoxic conditions (Fig. 4d). As expected in the asynchronous setting, hypoxic cultures differed from normoxic cultures by having a higher proportion of cells in G1 phase and a lower proportion in S phase. Following infection, E1A expression was enhanced to a similar extent in synchronized cells arrested in G1 phase under both normoxia and hypoxia, compared with asynchronous cells (Fig. 4e). In cells released from G1-phase arrest, characterized by a G1-S-phase phenotype (Fig. 4d), infection resulted in E1A upregulation in normoxia but not in hypoxia, compared with asynchronous controls. Despite the E1A upregulation observed in synchronized cultures, G1-phase arrest of normoxic cells did not lead to the elevated E1A protein levels observed in hypoxia. This suggests that hypoxia-induced G1-phase accumulation contributed, to a limited extent, to the observed E1A upregulation. Notably, when monitoring hypoxic signaling in this experiment, HIF-2α levels were elevated in hypoxic EnAd-infected cells compared with mock-infected controls.

### Role of HIF in transactivation of *E1A*

**Fig. 5:**
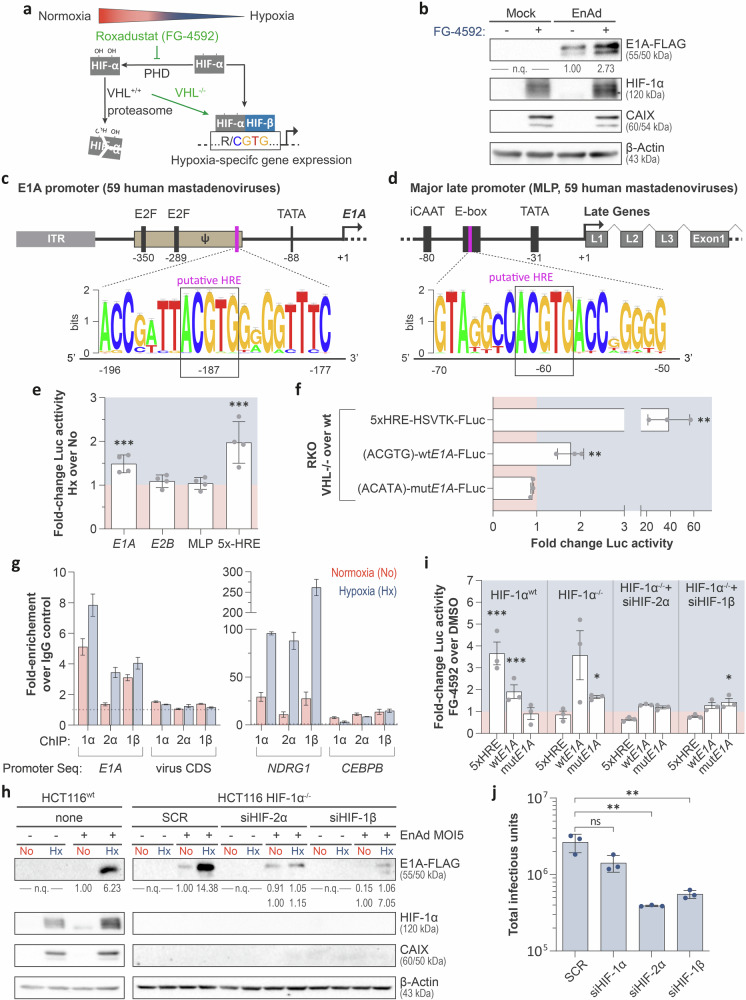
The *E1A* promoter is activated by hypoxia and constitutive HIF signaling through a conserved HRE involving HIF-2α and HIF-1β binding. **a** Schematic of the hypoxia-inducible factor (HIF) pathway highlighting HIF-stabilization by knockout of Von–Hippel–Lindau (VHL) and by inhibition of prolyl-hydroxylase domain protein (PHD) with FG-4592, adapted from Yousaf et al.^12^ (licensed under CC BY 4.0). **b** DLD-1 cells treated with FG-4592 were infected with enadenotucirev (EnAd-E1A-cFLAG) at a multiplicity of infection of 5 (MOI) and harvested at 8 h post-infection (hpi) for immunoblotting of FLAG-tagged E1A, HIF-1α, CAIX (HIF-1α target), and β-Actin. **c**, **d** The *E1A* and major late promoter genomic regions are shown schematically, with a sequence logo indicating the conservation frequency of putative R/CGTG hypoxia-response elements (HRE) in 59 human mastadenoviruses (species A-G); highlighted elements are: 5´-inverted terminal repeat (ITR), the packaging signal (ψ), E2F-binding sites, regulatory motifs (TATA, E-box, and inverted CAAT box), and leader sequences 1 to 3 (L1-3). **e** Induction of the EnAd/Ad11p-derived *E1A*, *E2B*, and MLP promoters in DLD-1 cells was compared under hypoxia and normoxia using luciferase reporter plasmids; a 5xHRE reporter served as a positive control, *n* = 4. **f** Activity of the HRE-mutant E1A promoter (ACGTG-to-ACATA, mut*E1A*) and the wild-type promoter (wt*E1A*) was compared between VHL^−/−^ and wild-type RKO cells, *n* = 3. **g** Enrichment of *E1A* (99-bp region encompassing the putative HRE), *NDRG1* (positive control), and negative control sequences (*CEBPB* promoter and viral coding-sequence, CDS) was measured by chromatin immunoprecipitation ChIP-qPCR using HIF-1α (1α), HIF-2α (2α), and HIF-1β (1β) antibodies in SK-OV-3 cells pre-exposed to normoxia or hypoxia for 18 h and infected at MOI = 20 for 5 h; technical replicates of a representative experiment. **h** HCT116^wt^ or HIF-1α^−/–^ cells transfected with scramble control (SCR), HIF-2α, or HIF-1β siRNAs were either pre-exposed to normoxia (No) or hypoxia (Hx), infected at MOI = 3, and harvested 8 hpi for immunoblotting; or **i** transfected with wt*E1A* or mut*E1A* reporters and treated with FG-4592 for promoter activity analysis, *n* = 3. **j** Virus production in SK-OV-3 cells under hypoxia was measured following transfection with either SCR, HIF-1α, HIF-2α, or HIF-1β siRNAs and infection with EnAd-SA-fLuc at MOI 10, *n* = 3. Data are shown as mean ± SD, *P* value: not significant (ns) *P* > 0.05, *P ≤ 0.05, **P ≤ 0.01, ***P ≤ 0.001. Negative control bands were not quantified (n.q.)

Next, we investigated whether HIF transcription factors enhance E1A expression under pharmacological hypoxia induced by the PHD inhibitor FG-4592. This molecule prevents PHD-mediated hydroxylation of HIFs, thereby mimicking hypoxia^28^ (Fig. 5a), as confirmed by the stabilization of HIF-1α under normoxia (Fig. 5b). In EnAd-E1A-cFLAG infected cells, FG-4592 treatment increased E1A expression in normoxia to levels comparable with those seen under low oxygen conditions (Fig. 5b).

Because pharmacological hypoxia alone upregulated E1A, we suspected that HIF transcription factors directly interact with viral promoters. Scanning the Ad11p viral genome revealed ACGTG motifs in the *E1A* and MLP promoters that matched the putative HRE (R/CGTG).^20^ The sequence was highly conserved across the 59 analyzed human mastadenoviruses, spanning all seven species (Fig. 5c, d and Supplementary Table 1). The HREs mapped to positions −187 bp upstream of the *E1A* transcription start site within the packaging signal and to −60 bp upstream of a stimulatory element in the MLP, which contains an E-box binding site for the Upstream transcription factor (USF).^29^

To assess the impact of hypoxia on these promoters, we used luciferase reporters containing the *E1A*, *E2B*, and MLP promoters. Under hypoxia, both the *E1A* reporter and an artificial positive control comprising a 5× tandem HRE repeat and the herpes simplex virus thymidine kinase minimal promoter showed increased activity relative to normoxia (~50% and ~90%, respectively, Fig. 5e). In contrast, the *E2B* and MLP reporters were not induced under hypoxic conditions. To confirm that hypoxia-specific *E1A* induction occurs via the identified HRE site, we mutated the ACGTG site in the *E1A* reporter plasmid to ACATA. Constitutive HIF-stabilization in VHL^−/−^ RKO cells induced the wild-type *E1A* promoter activity 1.8-fold, with no effect on the HRE mutant version, confirming the functionality of the ACGTG site and suggesting direct HIF binding (Fig. 5f). *E1A* promoter sequences covering the identified HRE were enriched in chromatin immunoprecipitation assays for HIF-1α, HIF-2α, and HIF-1β (Fig. 5g). This was validated by the absence of non-promoter viral coding sequences, low HIF occupancy in the host CEBPB promoter region, and high HIF occupancy in the NRDG1 promoter region, as previously reported.^30^

Since all HIF subunits associated with the *E1A* promoter chromatin to a certain extent, we proceeded to identify which subunit leads to functional upregulation of *E1A* under hypoxia. To this end, HIF-1α-knockout HCT116 cells were transfected with non-targeting siRNAs or siRNAs against HIF-2α or HIF-1β, followed by infection in normoxia or hypoxia. Hypoxia-induced upregulation of E1A was suppressed upon knockdown of HIF-2α or HIF-1β, yet persisted in non-targeting siRNA and HCT116 wild-type controls (Fig. 5h). Similarly, using the wild-type and mutant E1A reporters under pharmacological hypoxia, induction of the wild-type *E1A* promoter was abrogated by depletion of HIF-2α or HIF-1β, but not by HIF-1α knockdown alone, whereas induction was absent in the mutant reporter (Fig. 5i). This indicates that HIF-2α and HIF-1β, but not HIF-1α, play a role in regulating *E1A* levels. Of note, EnAd infection led to minor HIF-1α induction in normoxia in HCT116^wt^ cells (Fig. 5h), which was not observed in other cancer cell lines used in this study.

To further demonstrate the role of the HRE in the *E1A* promoter, we set out to generate a virus with a mutated HRE, as described for the *E1A* locus manipulation in Supplementary Fig. 4. Despite several attempts, the virus could not be rescued after transfection, potentially due to the proximity of the mutated HRE to the viral packaging signal (ψ, Fig. 5c). However, depleting HIF-2α or HIF-1β, but not HIF-1α, reduced infectious virus titers eightfold under hypoxia, further supporting the involvement of HIF-2α and HIF-1β in *E1A* transcriptional activation independent of HIF-1α (Fig. 5j).

## Discussion

### Hypoxia-driven enhancement of adenovirus replication

**Fig. 6:**
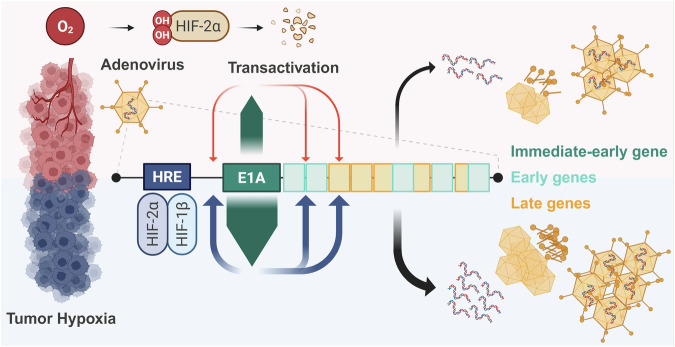
Hypoxia-driven enhancement of oncolytic adenoviral activity via HIF-2α and HIF-1β-dependent transactivation of *E1A*. In a synchronous infection under hypoxic conditions, mimicking a feature of the tumor microenvironment, HIF-2α and HIF-1β heterodimers bind a conserved hypoxia response element (HRE) within the adenovirus *E1A* promoter. This drives upregulation of the E1A transactivator in cancer cells. Elevated E1A under hypoxia enhances expression of immediate-early, early, and late virus genes, thereby boosting the adenoviral life cycle, including genome replication, structural protein production, virion assembly, and virus burst. The HRE is widely conserved across mastadenovirus genotypes, and this mechanism was demonstrated for the clinically relevant chimeric group B adenovirus (Ad11p/Ad3), enadenotucirev. Illustration was created in BioRender and licensed under CC BY 4.0 (https://BioRender.com/wnqmd2g)

Our study on the effects of hypoxia on the clinically relevant group B adenovirus EnAd revealed higher virus prevalence in hypoxic tumor regions in vivo and increased virus production in multiple cancer cell lines cultured under hypoxia. During a single-infection cycle, hypoxia upregulated viral transcription and translation, particularly accelerating and boosting expression of the immediate-early gene *E1A*. Concomitantly, genome synthesis and late viral gene expression were elevated, thereby sustaining enhanced EnAd production, which was also observed with the group C adenovirus Ad5. A putative and functional HRE in the *E1A* promoter region was identified. Knockdown studies implicated *E1A* induction via HIF-2α/1β heterodimers as a key driver of increased viral activity under hypoxia, providing a rationale for the increased prevalence of infection in hypoxic areas. The conservation of the HRE across 59 human mastadenovirus species suggests that hypoxia-mediated control is likely widespread across adenoviruses (Fig. 6).

### Viral mechanisms underlying enhanced virus production in hypoxia

Hypoxia enhanced infectious virion production without affecting viral entry, infection cycle kinetics, genome packaging, or assembly of progeny particles. Instead, the increased viral burst size in hypoxia was triggered by elevated transcription and translation of *E1A*, the first viral gene induced upon infection. E1A transactivates itself and other viral and host genes via positive feedback,^24^ as evidenced in EnAd by the sequential induction of the E2B polymerase and virus genome accumulation, and in Ad5 by increased genome replication. Elevated E2B levels likely support greater production of infectious particles under hypoxia, as its abundance is critical for adenovirus burst size.^31^ Furthermore, late virus genes driven by the MLP were enhanced under hypoxia, supporting structural protein synthesis necessary for virion assembly. This aligns with evidence that Ad5 E1A enhances MLP activity,^32^ and suggests a similar role for E1A in group B adenoviruses. The effect on late transcription was transferable to the MLP-coupled bispecific antibody, but remained modest, perhaps as its secretion approached saturation. The same payload, expressed from a CMV promoter in EnAd, was only slightly induced in hypoxia, possibly reflecting the reported oxygen sensitivity of the CMV promoter.^33^ Upregulation of viral proteins occurred despite a ~30% decline in global translation in hypoxia, when cap-independent translation and alternative ribosomal compositions sustain essential protein synthesis.^34^ Adenoviruses exploit the host´s cellular energetics to favor viral protein synthesis; for instance, 5’-leader sequences in MLP-derived transcripts enable ribosome shunting, a cap-independent translation mechanism.^35,36^ Moreover, the Ad5 100 K protein inhibits host cap-dependent translation, redirecting biosynthesis towards viral proteins.^37^ These mechanisms likely synergize to favor viral protein expression under hypoxia. Thus far, non-canonical translation of immediate-early genes has not been described, suggesting that E1A upregulation is driven primarily by increased promoter activity and mRNA synthesis. *E1A* overexpression in hypoxic cancer cells emerges as a driver of increased adenovirus group B (Ad11p/EnAd) and C (Ad5) production through enhanced early and late transcription, highlighting their potential to deliver virus-encoded biotherapeutics to hypoxic tumor regions.

### Cellular mechanisms shaping adenovirus replication in hypoxia

Despite reduced global mRNA synthesis in hypoxic cells,^12,38^ viral genes were still upregulated, indicating virus-specific gene induction. In examining known mechanisms underlying *E1A* induction, we concluded that neither reduced DAXX-mediated transcriptional suppression nor G1-arrest explained the hypoxia-driven E1A upregulation. Although hypoxic EnAd-infected cells inactivate DAXX more efficiently via cytoplasmic mobilization, the difference in E1A expression and virus production between hypoxia and normoxia was DAXX-independent. While DAXX depletion is known to contribute to G1-arrest,^39^ forcing normoxic cells into G1-arrest did not replicate the hypoxia-driven E1A overexpression; instead, it increased E1A expression under both oxygen conditions. DAXX knockdown significantly boosted virus yield (>50-fold) and revealed an underappreciated virus-sensitizing approach relevant to adenovirus manufacturing. Our findings in hypoxia complement prior studies in normoxia showing G1phase-dependent induction of E1A,^40^ and confirm the link between DAXX abrogation and viral replication.^41^

In turn, pharmacological HIF stabilization under normoxic conditions did induce E1A to levels similar to those observed in hypoxia, implicating the HIF system. A widely conserved HRE in the *E1A* promoter was confirmed to be hypoxia-inducible and was occupied by HIF transcription factors to an extent similar to that reported for HREs in other virus families.^42^ HRE-adjacent sequences and additional transcription factors may cooperate with HIF to induce the *E1A* promoter, as a putative HRE identified in the MLP promoter was nonfunctional based on reporter assays. The functional HRE was sensitive to HIF-2α and HIF-1β knockdowns, but less sensitive to HIF-1α knockout, suggesting transcriptional activation predominantly via HIF-2α/1β heterodimers. While HRE-like sequences were previously noted in Ad5,^43^ this is the first demonstration of a functional HRE in a group B adenovirus (Ad11p/EnAd), substantiated by converging results from promoter occupancy studies, mutant constructs, siRNA knockdowns, use of multiple cell lines, and ultimately by demonstrating reduced virus production after HIF-2α silencing. The *E1A* HRE, conserved across mastadenoviruses, appears to be critical for adenoviral fitness, possibly due to its overlap with the virus packaging signal, though its role in pathology remains unclear. Functional HREs also exist in Epstein-Barr virus, hepatitis B virus, and even aquatic viruses, suggesting hypoxia-specific viral gene expression as a common evolutionary trait.^42,44,45^ As for the design of oncolytic adenoviruses, our study indicates that adenoviruses are already equipped to thrive in hypoxic conditions without the need for synthetic hypoxia-specific promoters.

Notably, early after infection, HIF-2α —but not HIF-1α— was overexpressed in both hypoxia and normoxia. We previously reported that this is caused by HIF-2α mRNA induction early in viral infection, independent of oxygen tension, followed by attenuation of HIF signaling during the late viral phase in a VHL/PHD-independent manner.^12^ Because changes in HIF expression during the viral life cycle are isoform-specific and VHL/PHD-independent, EnAd is unlikely to be equipped to override the oxygen-sensing machinery to stabilize HIF. Increased HIF signaling early during infection under hypoxia may cooperate to enhance viral activity, not only through the activation of the E1A promoter reported herein but also via HIF-triggered metabolic reprogramming. For instance, HIF signaling induces anabolic pathways such as reductive carboxylation,^46,47^ which EnAd exploits to sustain replication.^48^ Despite these additional contributions, E1A expression remains a key driver of viral activity, as evidenced by substantial increases in virus production following modest increases in E1A protein levels after DAXX silencing under both hypoxic and normoxic conditions.

### Activity of oncolytic adenoviruses under hypoxia

Our findings challenge previous reports of reduced E1A expression and oncolytic activity under hypoxia.^15,16,49^ These discrepancies likely reflect differences in experimental conditions. Maintaining prolonged low-oxygen conditions in plastic culture dishes is challenging because cancer cells quickly deplete dissolved oxygen, becoming severely hypoxic or anoxic. To address this, we used gas-permeable plates and pre-conditioned cells at a constant 1% pO_2_ (mild hypoxia). While earlier studies examined multi-step infections over several days, we focused on synchronous, high-MOI infections to isolate hypoxia’s direct effects on specific stages of the viral cycle. This approach mitigates the metabolic constraints of prolonged culture and the effects of hypoxia-associated antiviral signaling, as synchronous adenovirus infection is largely refractory to activation of antiviral pathways (e.g., TBK1/IRF3) and to the concomitant secretion of type-I interferons.^50^ Only a few hypoxia studies at high-MOI exist, and they report increased *E1A* transcription for Ad5 and increased overall oncolytic activity for HSV.^43,51^ Our study confirms that EnAd completes its life cycle under both high- and low-oxygen conditions, suggesting its intrinsic ability to target tumors across oxygen gradients.

Previously, we showed that systemic EnAd administration results in infection of both hypoxic and well-oxygenated tumor regions and that infected nodules exhibit reduced vessel perfusion.^12^ Here, we extended this observation by demonstrating that EnAd infection foci are, in fact, enriched in non-necrotic regions experiencing low oxygen. This spatial distribution likely reflects the hypoxia sensitivity of *E1A* transcription, which drives viral transcription and translation to enhance virus production, and may underpin anticancer activity in hypoxic tumor compartments. Oncoselectivity is presumably maintained even under hypoxic conditions, as non-transformed cells, such as stromal fibroblasts, are highly resistant to EnAd infection.^8,23^ Our findings are of clinical interest and might provide context for the encouraging results observed in patients treated with EnAd plus chemoradiation (CEDAR trial, NCT0391510).^52,53^ This trial enabled further clinical testing of a next-generation candidate armed with a therapeutic transgene (FORTRESS trial, NCT06459869). Collectively, the positive effects of hypoxia on viral activity described herein, the previously reported impact of EnAd on tumor architecture,^12^ and the dynamic changes in tumor perfusion that drive local oxygen and metabolic shifts and alter antiviral signaling warrant further investigation into the ability of oncolytic adenoviruses to sustain activity as they spread through oxygen gradients.

In conclusion, our findings identify a positive role for hypoxia in oncolytic adenovirus production through HIF-2α/1β-driven activation of the *E1A* promoter, enhancing viral transcription via positive feedback. This study provides the first demonstration of a functional HRE in an adenovirus and offers new insights into group B adenovirus infection in low-oxygen environments.

## Materials and methods

### Spatial proximity analysis of adenovirus foci and hypoxia

Animal experimentation was conducted under ethical approval from the UK Home Office (Project license 30/3391 and personal license I5916669E). Female CB17-SCID mice (6–8 weeks old) were inoculated subcutaneously in the right flank with 2 × 10^6^ DLD-1 cells in 50 µL. On day 1, when xenografts reached 80–120 mm^3^, mice received 100 µL of chlodronate liposomes (Chlodronateliposomes.org) intravenously to maximize subsequent adenovirus delivery.^54^ EnAd-SA-fLuc reporter virus particles (2 × 10^10^) were injected via the tail vein on days 2 and 4. During steady-state infection, previously shown to be 16 days after the last treatment,^12^ mice were injected intraperitoneally with pimonidazole at 60 mg·kg^−1^ and were culled 2 h later. Tumors were explanted, formalin-fixed, and paraffin-embedded.

Eight pairs of serial tissue sections per tumor (4 µm thick, ca. 40 µm apart, 4 pairs for mouse 3) were stained for hexon (horseradish peroxidase/3,3´-diaminobenzidine), pimonidazole adducts (alkaline phosphatase/Fast red), and nuclei (hematoxylin) as previously described.^12^ Spatial proximity between hexon-positive adenovirus foci and hypoxic regions was analyzed using a custom pipeline in Fiji/ImageJ.^55^ Briefly, matched serial sections were scanned at 20x (Aperio ImageScope, Leica), aligned, cropped to tissue boundaries, and cleaned of staining artifacts. Binary masks were generated from color-deconvolved signals using intensity thresholding. The pimonidazole mask was skeletonized to render an Euclidean distance map extending 200 µm outward from the core/skeleton of pimonidazole staining. Hexon- and hematoxylin-positive areas, representing nuclei in viable tumor regions, were projected onto this map to extract the normalized pixel fraction as a function of distance from the pimonidazole skeleton. A detailed framework is provided in Supplementary Fig. 1.

### Cell lines

DLD-1, SW480, SK-OV-3, HCT116, HT29 (ATCC), and AD293 (Agilent Technologies) were cultured in DMEM (Sigma Aldrich) with 10% (v/v) heat-inactivated fetal bovine serum (FBS, Gibco) at 37 °C and 5% CO_2._ Cell lines were maintained in a humidified incubator, routinely tested for mycoplasma (Lonza), and authenticated (Source Bioscience).

### Hypoxia treatment

Hypoxia incubation (1% pO_2_) was performed in the In vivo2 400 chamber (Baker Ruskin) using media pre-equilibrated overnight at 1% pO₂. Experiments utilized gas-permeable Lumox® plates (Sarstedt) on a zig-zag rocker (PMR-30, VWR) at five cycles per minute to maintain gas equilibrium. External oxygen calibration of the chamber was performed routinely. Pharmacological hypoxia was induced with 50 µM FG4592 (Roxadustat, Cayman Chemical).

### Viruses and infections

EnAd-E1A-cFLAG, used for E1A expression analysis, was generated using a shuttle plasmid containing the *E1A* open reading frame flanked by *Nde*I sites in the pColoAd2.4 vector.^23^ Cloning of this virus is summarized in Supplementary Figs. 3 and 4. EnAd-SA-fLuc, used in experiments tracking viral life cycle, contains a firefly luciferase coupled to the major late transcription unit by a splice acceptor sequence.^23^ EnAd-CMV-BiTE and EnAd-SA-BiTE express a deca-His-tagged EpCAM/CD3-BiTE under a CMV promoter or integrated into the late transcription unit via a splice acceptor, respectively.^25^ Ad5-E1A-fLuc is a replication-competent reporter adenovirus encoding firefly luciferase fused to E1A.^56^ Virus stocks were concentrated and double-purified via cesium chloride gradient centrifugation, then titered by plaque assay on A549 cells as previously described.^12^

Infections were performed in serum-free media for 2 h. For virus production, inocula were removed, and cells were washed twice with complete medium. The inoculum was set to 125 μL·cm^−2^ independent of the seeding format, and MOIs were based on titers derived by plaque assay (Supplementary Table 2).

### Quantification of infectious adenovirus particles using bioluminescence

A high-dynamic-range method was established to measure infectious viral particles by correlating light emitted by reporter adenoviruses of unknown concentration with plaque assay-tittered standards (Supplementary Fig. 2). Phenol red-free supernatants from virus production assays with EnAd-SA-fLuc were collected, weighed, and cleared by centrifugation. Neat or diluted 25 µL samples, along with a standard series (5.57 × 10^5^ PFU·mL^−1^ 2.72 × 10^2^ PFU·mL^−1^), were used to infect 1.5 × 10^4^ A549 cells seeded the previous day in 50 µL phenol red-free DMEM + 10% FBS (Gibco) on solid black 96-well plates (Corning). Plates were then spun at 600×*g*_n_ for 10 min. At 20 to 24 hpi, luminescence was measured using a PolarStar plate reader (BMG Labtech) after injecting 25 µL of a 1.2 mg·mL^−1^ D-Luciferin solution into the well (Gold Biotechnology). Bioluminescence was recorded over six kinetic cycles at 0.5 s intervals, and virus concentrations were calculated via four-parameter fitting of integrated luminescence (MARS, BMG Labtech). At least three technical replicates were performed for each infection.

### Assay for capsid integrity

To analyze the protein composition of adenovirus particles in supernatants, the culture medium was centrifuged (300×*g*_n_, 5 min), filtered (0.22 µm), and concentrated using a 300 kDa cut-off ultrafiltration unit (Sartorius). The retentate was washed twice with PBS, and protein concentration was measured using the QuantiPro BCA Kit (Thermo Fisher). Two micrograms of lysate in RIPA buffer (Thermo Fisher) were separated as for immunoblotting, stained with SyproRuby (Thermo Fisher) and imaged on a Biorad Chemidoc imager.

### Absolute quantification of viral mRNA and genomic DNA by qPCR

DNA was extracted from infected cell pellets or supernatants using the PureLink genomic DNA Mini Kit (Invitrogen), and total RNA using RNeasy Mini Kit (Qiagen). RNA integrity was routinely tested by capillary electrophoresis (Tape Station, Agilent). For cDNA synthesis, 500 to 800 ng of RNA was transcribed using the QuantiTect Kit (Qiagen). PCR reactions (20 µL) contained 10 ng of cDNA or DNA with 2x qPCRBIO Probe Mix Hi-ROX (TaqMan assays, PCR Biosystems). *Fiber*, *E1A*, and *E2B* 200 bp-standards for absolute quantification were synthesized (IDT Technologies, Supplementary Fig. 7). *Fiber* detection was used to quantify genome copy number. TaqMan probes were labeled with JOE or FAM and quenched with BHQ1 (Sigma). Measurements were performed on the StepOnePlus cycler (Applied Biosciences). Copy numbers were calculated by linear regression on standard curves (Supplementary Fig. 7). Primer sequences are available in Supplementary Table 3.

To quantify encapsidated genomes, 200 µL of infected supernatants were treated with 100U Benzonase (Millipore) for 3 h at 37 °C. Before DNA extraction and fiber quantification by qPCR, benzonase was inactivated with EDTA (0.1 M final concentration) and heat-inactivation at 80 °C for 10 min.

### Flow cytometry

Cells were detached using cell dissociation buffer (GIBCO), stained for 10 min with LIVE/DEAD fixable near-infrared staining kit (1:5000, Thermo Fisher), and then fixed in 2% neutral-buffered formalin for 10 min (Sigma). Antigens were blocked in 2 mM EDTA + 0.5% BSA with Fc-block (1:100, Biolegend) in PBS for 20 min. Cells were then stained with anti-CD46 antibody (1:200, clone TRA-2-10, Biolegend) or the corresponding isotype control for 30 min in PBS + 2 mM EDTA + 0.5% BSA. Stained cells were measured on the FACSCalibur flow cytometer (BD Biosciences). Analysis was performed on cell populations excluding debris, doublets and dead cells.

### Real-time monitoring of cytopathic effect

Loss of cell adhesion due to cytopathic effect was monitored in real-time using the xCELLigence RTCA DP instrument (Roche). xCELLigence plates were equilibrated with 50 µL of medium for 1 h to establish a baseline. Then, 10,000 cells were plated in 100 µL of growth medium. Infections were initiated with 50 µL, and impedance (cell index) was recorded every 15 min. Measurements were normalized 4 h before infection.

### Immunoblotting

Immunoblotting was performed as described previously, with blot development on X-ray films (GE Healthcare) or the Biorad Chemidoc imager.^12^ Dot blots were performed to quantify the secretion of deca-His-tagged EpCAM/CD3-BiTE, as described previously.^57^ Nuclear and cytoplasmic fractionation was performed using the NE-PER™ Kit according to the manufacturer’s instructions (Thermo Scientific). Antibody dilutions and blocking conditions are specified in Supplementary Table 4. Semi-quantitative assessment of band intensity was performed by densitometry using the ImageLab software (Biorad), with intensity normalization to the loading control of the same run.

### Cell cycle synchronization and analysis

Cell synchronization was achieved using a double thymidine block: 2 mM thymidine (Sigma) was added for 16 h, followed by an 8-h release, and another 16-h block. For cell cycle analysis, cells were pulsed with 20 µM bromodeoxyuridine (BrdU, Sigma) for 10 min before harvest. Cells were subsequently detached using trypsin, fixed in ice-cold 70% ethanol for 30 min, and treated with 2 M HCl + 1 mg·mL^−1^ pepsin (Sigma) for 20 min. After two PBS washes, cells were stained with a primary mouse anti-BrdU antibody (1:100, 90 min, BD Biosciences) and a secondary goat anti-mouse-488 Fab-fragment (1:500, 60 min, Thermo Fisher) in DPBS + 2% FBS. After washing, cells were stained with 50 µg·mL^−1^ propidium iodide and 400 µg·mL^−1^ RNase A. Flow cytometry was performed on an Attune flow cytometer (Thermo Fisher), and data were processed with FlowJo v10.0.7r2 software (TreeStar Inc., USA).

### Assay for promoter activity

In total, 5 × 10⁴ cells were seeded in 24-well plates. The next day, 200 ng of plasmid per reaction (~600–800 pmol) and 2.5 µL Lipofectamine 2000 per 1000 ng DNA were each diluted in 150 µL Optimem (Thermo Fisher), combined, and incubated for 30 min before adding to cells. After overnight incubation, the medium was replaced to start hypoxic exposure. Co-transfection was performed with one twentieth of the transfected plasmid mass of a β-galactosidase-encoding plasmid (SV40-betaGal, Promega) to normalize for transfection efficiency. Cells were washed with PBS, lysed, and subjected to one freeze-thaw cycle before measuring luciferase activity in black 96-well plates with luciferin reagent (Promega). Luciferase expression was normalized to β-galactosidase activity, which was measured by incubating lysates with 2× ONPG buffer (137 mM HNa_2_PO_4_ + 63 mM H_2_NaPO_4_ + 2 mM MgCl_2_ + 0.7% (v/v) β-mercaptoethanol + 1.33 mg∙mL^−1^ ortho-Nitrophenyl-β-galactoside, pH 7.2) at 37 °C for 10 min. Reactions were stopped with 1 M Na₂CO₃, and OD was measured at 410 nm.

### Puromycin incorporation assay

Global translation was assessed by pulsing cells with 1 µg∙mL^−1^ puromycin (Sigma) for 30 min. Pre-treatment with cycloheximide (100 µM) served as a negative control. Lysates were then subjected to immunoblotting.

### Reverse transfection of siRNA

siRNA transfections were conducted at a ratio of 10 pmol siRNA to 1 µL RNAiMax (Thermo Fisher) in Optimem (Thermo Fisher). After mixing, the solutions were incubated for 30 min before plating 3 × 10⁴ cells (24-well) or 1.2 × 10⁵ cells (6-well). After overnight incubation, the medium was replaced with complete medium to start hypoxic treatment. siRNA sequences are listed in Supplementary Table 5.

### Sequence alignment of adenovirus genomes

Putative HREs were identified by manual scanning of annotated promoters of Ad11p (GenBank: AY598970.1). Corresponding regions from 59 adenovirus genomes (Supplementary Table 1) were aligned using the CLC Genomics Workbench (Qiagen). Nucleotide frequencies of the HRE motif across all genomes were visualized as a sequence logo.

### Chromatin immunoprecipitation-qPCR (ChIP-qPCR)

In total, 6 × 10^6^ cells were plated onto 15-cm plates. Chromatin crosslinking was done with 1% methanol-free formaldehyde (v/v) for 10 min with gentle rocking, followed by quenching with 125 mM glycine for 10 min. Cells were washed twice on ice with PBS, scraped into 5 mL of PBS, and pelleted at 500× *g*. Pellets were resuspended in SDS lysis buffer (1% SDS, 10 mM EDTA, 50 mM Tris pH 8.1) with protease inhibitors (1:30, Thermo Fisher).

Sonication was performed at 4 °C using a Bioruptor (Diagenode) for 2:45 min (15-second pulses on/off, high power) followed by benzonase treatment (15 U, Milipore) for 25 min for adequate fragmentation of viral and host chromatin. DNA fragmentation (~300 bp) was confirmed via TapeStation. Protein A agarose beads (40 µL, Millipore) were pre-washed with ChIP diluent and then incubated with sonicated DNA for 1 h at 4 °C on an end-over-end rotator. As previously described,^58^ 15 µL of HIF-1α (PM14), HIF-2α (PM9), HIF-1β (Novus NB100-110), or pre-immune sera (10 µL) were used per ChIP reaction and incubated overnight at 4 °C. After bead addition (90 µL), samples were incubated for 1.5 h at 4 °C with end-to-end rotation, and then pelleted at 380×*g*_n_ for 8 min. Washes (800 µL) were performed with low- and high-salt buffers and LiCl buffers at 4 °C for 5 min each. The samples were pelleted at 380×*g*_n_ at 4 °C for 5 min, then washed twice in 800 µL TE. A two-step elution (120 µL buffer each step) was performed at room temperature for 15 min with shaking at 1400 rpm. For ChIP buffer components refer to Supplementary Table 7.

Reverse crosslinking was achieved by adding 12.5 µL of 4 M NaCl and heating at 65 °C overnight. Proteinase K (2 µl of 20 mg·mL^−1^) was added for 4 h at 45 °C, followed by RNase A (1 µL) at 37 °C for 30 min. Precipitated DNA was purified using MinElute columns (Qiagen) and eluted in 20 µL of DNase-free water. qPCR was performed using the 2× SyGreenBlue Hi-ROX in 20 µL (PCR Biosystems), taking PCR efficiency into account (Supplementary Table 6).

### Statistical analysis

All statistical analyses and plots were performed using Prism v.10.6 (Graphpad). Replicates (*n*) representing biological variance are plotted as single data points, and technical replicates are indicated where relevant. Bars represent the mean ± standard deviation, unless otherwise stated. For statistical assessment, log-normal data were selected to allow the use of parametric tests. Two-sided unpaired *t* tests assuming unequal variance for conservative significance evaluation were performed (Welch´s correction). Holm–Šidák correction was applied for multiple comparisons.

## Supplementary information

Supplementary Information

## Data Availability

All data supporting the findings are included in the main article and its supplementary information files. Further information and resources are available from the corresponding authors upon reasonable request.
