# Supplementary material for: Hypoxia increases the activity of oncolytic adenoviruses through HIF-2α-stimulated E1A expression
Source: Signal Transduct Target Ther. 2026 Aug 11;11:320. doi: 10.1038/s41392-026-02895-y (PMC13457585; doi:10.1038/s41392-026-02895-y)
Supplement: Supplementary file 1 [file 41392_2026_2895_MOESM1_ESM.pdf]

Supplementary Materials for

Hypoxia increases the activity of oncolytic adenoviruses through HIF-2α-stimulated E1A expression

Egon J. Jacobus, Véronique N. Lafleur, Kerry D. Fisher, David R. Mole, Leonard W. Seymour

Correspondence to len.seymour@oncology.ox.ac.uk and egon.jacobus@gmail.com

This file includes :

Supplementary Figures S1 to S7

Tables S1 to S7

Original uncropped Western blot images

Supplementary Figures

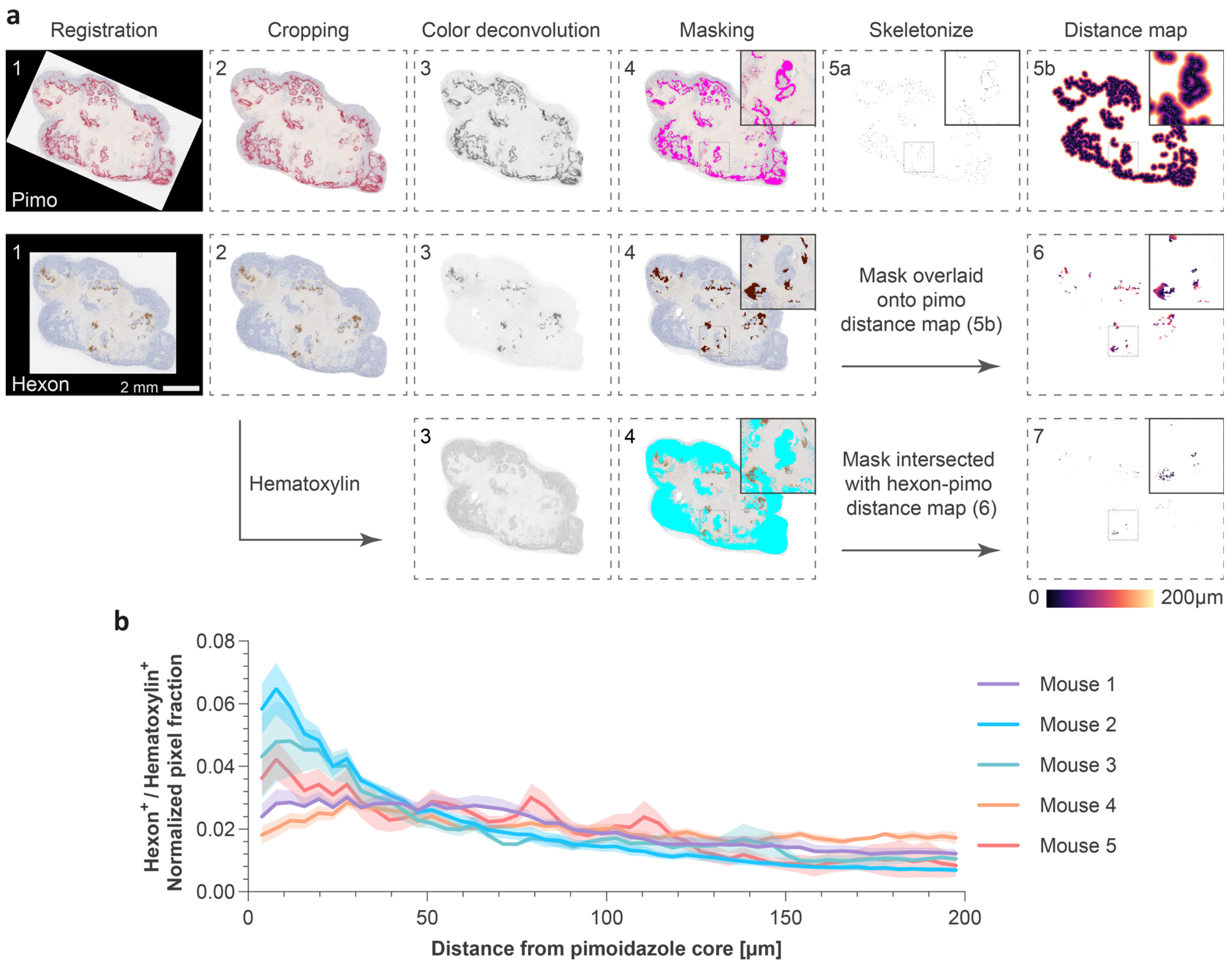

Supplementary Figure 1 Workflow for spatial proximity analysis between hexon-positive foci and hypoxic regions in histological sections. a Step1: Serial tissue sections stained for adenovirus hexon or pimonidazole adducts (Pimo) were aligned by image registration (20x magnification). Step2: Registered images were cropped to isolate tissue regions and exclude background. Step3: Color deconvolution was performed to separate signals from pimonidazole adducts (red), hexon (brown), or hematoxylin (blue). Step4: Binary masks were generated for all three images by user-defined intensity thresholding. Step5a: The pimonidazole mask was skeletonized to define the core of hypoxic, pimonidazole-positive regions. Step5b: Euclidean distance maps were computed from the skeletonized pimonidazole image, resulting in pixel values that report the distance from the core of hypoxic regions up to 200 µm, based on previously reported distances between pimonidazole staining, and perfused vessels[22]. Step6: Segmented hexon-positive foci were overlaid on the distance map. Step7: The hexon-pimonidazole distance map was then intersected with the hematoxylin-positive mask to exclude adenovirus foci in necrotic tissue. **b** Spatial proximity analysis of individual xenograft tumors. The mean (solid line) and standard deviation (colored area) of all analyzed images are shown.

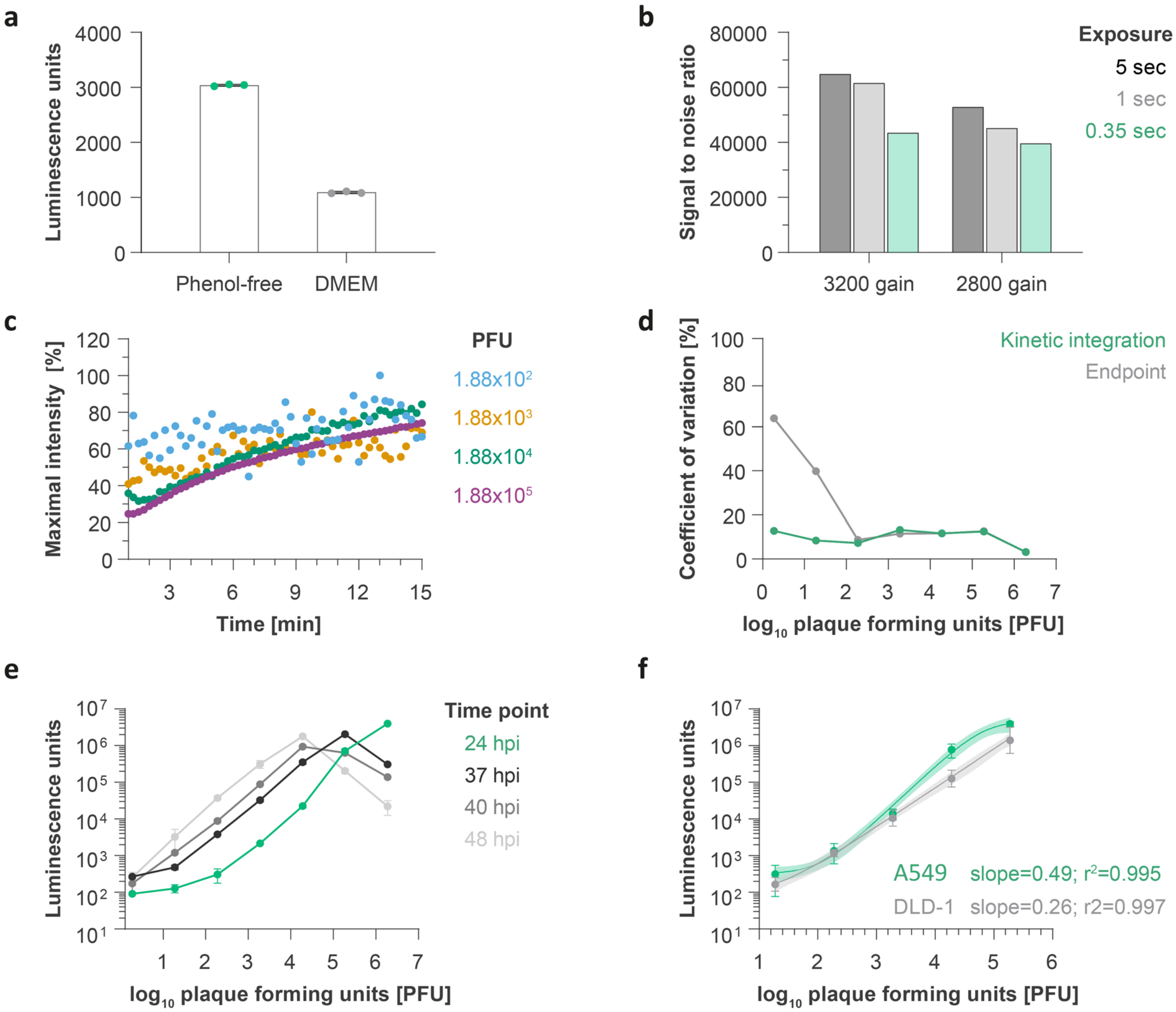

Supplementary Figure 2 Establishment of a luciferase-based assay for infectious particle quantification. **a** Luciferase signal after EnAd-SA-fLuc infection is shown in A549 cells cultured in DMEM with or without phenol red. **b** A sufficient signal-to-noise ratio was observed at 0.35-second exposure time for two gain levels, enabling short measurement times (single measurement). **c** Normalized maximal luminescence intensity in cells infected with varying amounts of EnAd-SA-fLuc plaque-forming units shows increased noise at low virus doses 24 hours post-infection (hpi) in an endpoint assay. **d** Mitigation of low signal-to-noise at low virus doses by kinetic integration of six measurement cycles yielded a low coefficient of variation compared with endpoint measurement over six logs. **e** The largest dynamic range in the measurement was observed at 24 hpi, two technical replicates. **f** With the optimizations from a-e (selected conditions in green), A549 cells were selected over DLD-1 cells because of higher signals. The signal from the EnAd-SA-fLuc dilution series was fitted with a four-parameter logistic model (error indicates the 95% CI). The assay range spans 20 to 2x10^{5} plaque-forming units in A549 cells, three orders of magnitude higher than that of the conventional plaque assay. Similar luciferase-based assays have been reported for vesicular stomatitis virus, herpes simplex virus 1, and vaccinia virus (Garcia et al., 2014). Single measurements or means of technical replicates ± SD are shown, unless otherwise indicated.

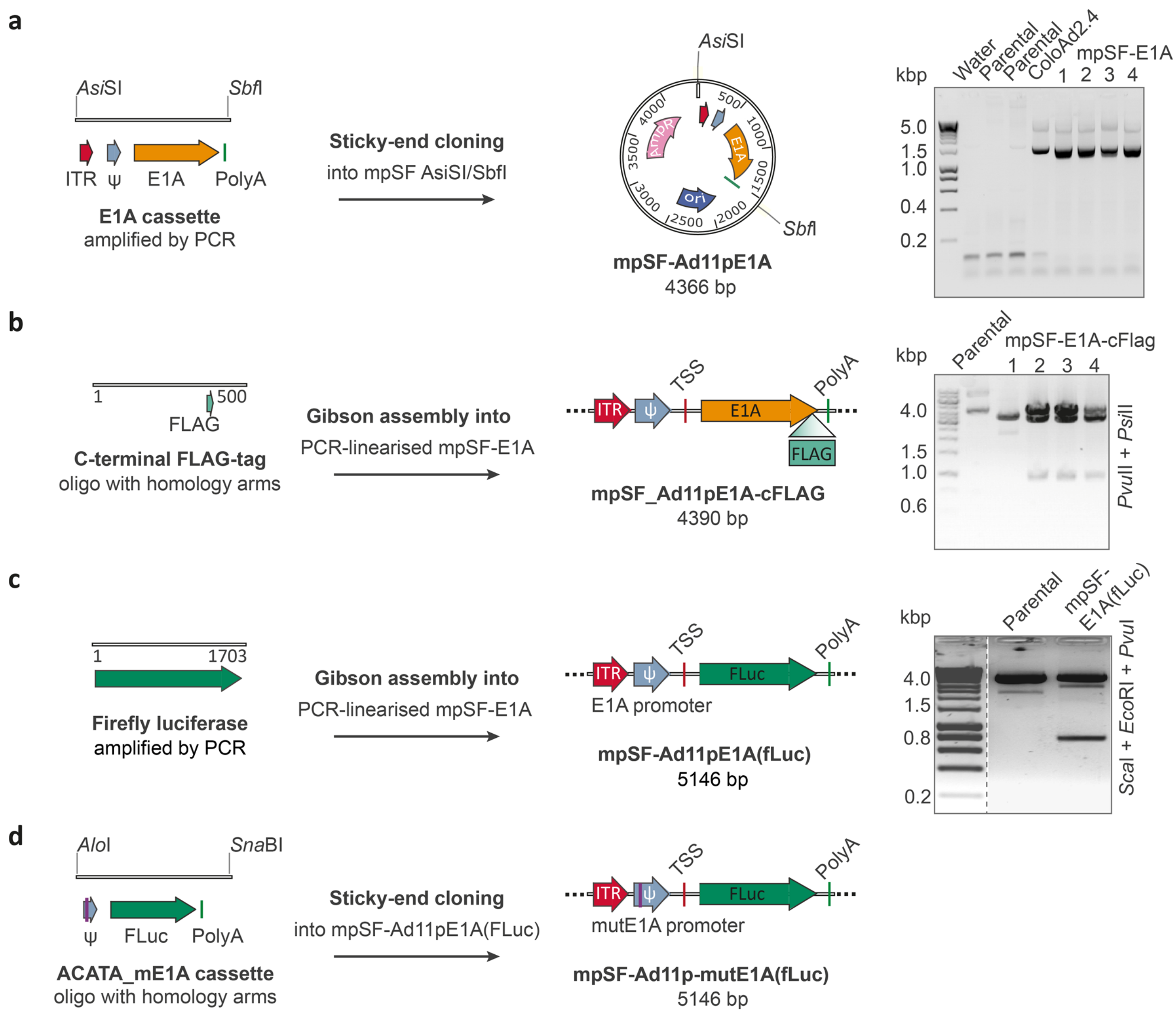

Supplementary Figure 3 Cloning of E1A reporter plasmids. a mpSF-E1A includes the 5' inverted terminal repeat (ITR), packaging signal (ψ), E1A promoter, transcription start site (TSS), ORF, and poly-A signal of EnAd. The E1A cassette was amplified by PCR and cloned into the mpSF plasmid via sticky-end ligation. Clones were screened by PCR, yielding a positive 1480 bp band. **b** A 500-bp dsDNA oligo with a FLAG tag at the C-terminus and homology arms was inserted into the linearized mpSF-E1A via PCR and cloned using Gibson assembly. Colonies were screened with HindIII and PsiI, yielding a positive 785-bp band. **c** Firefly luciferase (fLuc) was amplified by PCR with homology arms for mpSF-E1A and then ligated via Gibson assembly into linearized mpSF-E1A. Cloning was verified by ScaI, EcoRI, and PvuI, yielding a positive band at 764 bp. **d** A dsDNA oligo containing a mutant HRE sequence (ACATA) with overlapping arms was cloned into the reporter plasmid in C with *Alo*I and *Sna*BI.

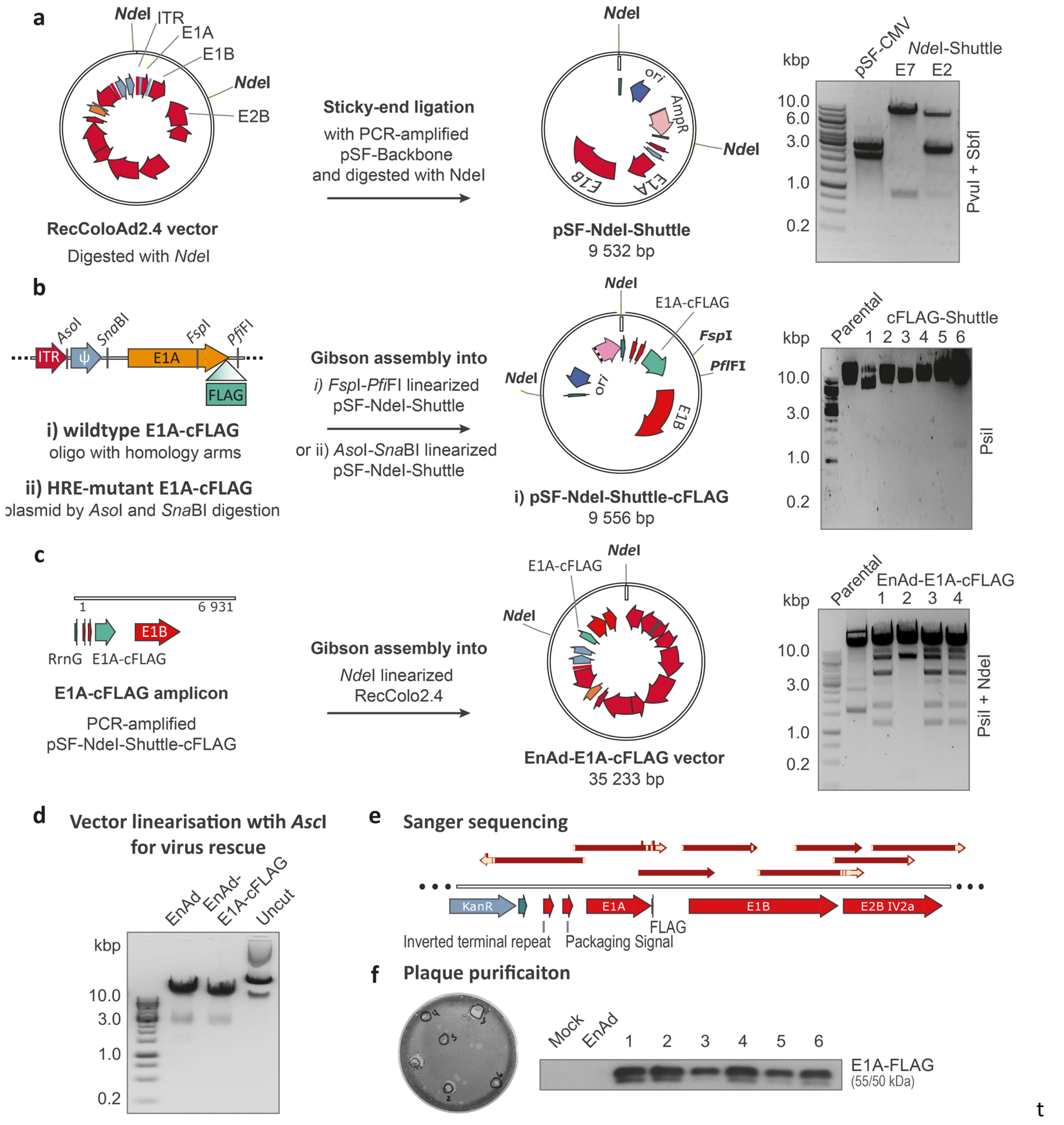

Supplementary Figure 4 Generation of EnAd-E1A-cFLAG virus. a The 6.6 kbp *Nde*I-flanked fragment from the RecColoAd2.4 plasmid was cloned into a pSF backbone via sticky-end cloning, producing pSF-NdeI-Shuttle. Clones were screened with *Sbf*I and *Pvu*I, yielding positive bands at 8655 and 877 bp (e.g., clone E7). **b** A dsDNA oligo with a FLAG-tag and homology arms for 3'-specific insertion into E1A or into the *Aso*I-*Sna*BI fragment of mpSF-Ad11p-HREmutE1A(fLuc) was cloned into a pSF-NdeI-Shuttle linearized with *Fsp*I-*Pfi*FI or *Aso*I-*Sna*BI, respectively. Cloning was confirmed using *Psi*I, resulting in bands at 8329 and 1227 bp (e.g., clone 6 for cFLAG Shuttle). **c** The modified NdeI-flanked fragment was PCR-amplified to introduce homology arms for ReColoAd2.4. The amplicon was ligated into an NdeI-

linearized RecColoAd2.4 backbone via Gibson assembly. Clones were screened with *Psi*I and *Nde*I, yielding bands at 1227, 1749, 3895, 6454, and 21908 bp (e.g., Clone 4 for EnAd-E1A-cFLAG). **d** Clone 4 was linearized with *Asc*I and purified for transfection into AD293 cells. EnAd-E1A-HREmut-cFLAg was also generated (as in b and c), but no replicating virus was rescued despite several attempts. **e** Clone 4 was confirmed by primer walking. **f** Five of six clones resulted in E1A-FLAG expression after the first round of plaque purification. Clone 2 was subjected to another round of plaque purification, propagation, and $CsCl_2$ purification.

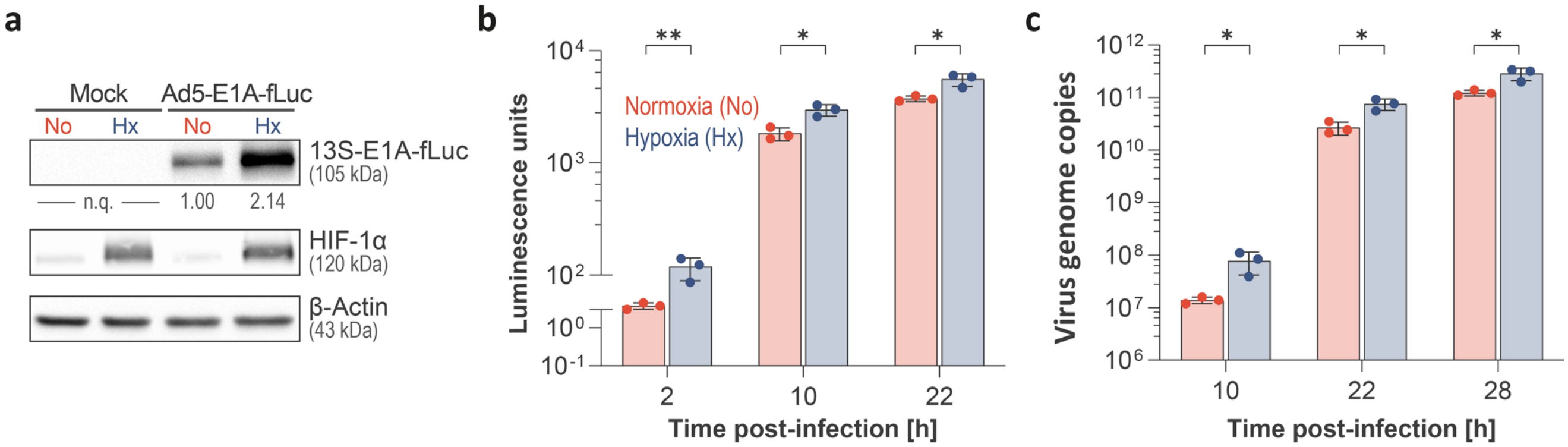

Supplementary Figure 5 Hypoxia enhances E1A expression and genome synthesis during Ad5 infection. DLD-1 cells were pre-exposed to hypoxia or normoxia for 18 hours, infected with Ad5-E1A-fLuc at MOI 5, and maintained at the respective oxygen tension throughout the infection. **a, b** E1A-fLuc protein expression was quantified by immunoblotting 8 hours post-infection, and over time via bioluminescence detection of the E1A-luciferase fusion, n = 3. **c** Ad5-E1A-fLuc genome synthesis was measured by qPCR, n = 3. Data are shown as mean ± SD, p-value: $p > 0.05$: not significant (ns), $p \leq 0.05$: *, $p \leq 0.01$ **, $p \leq 0.001$: ***.Negative control bands were not quantified (n.q.).

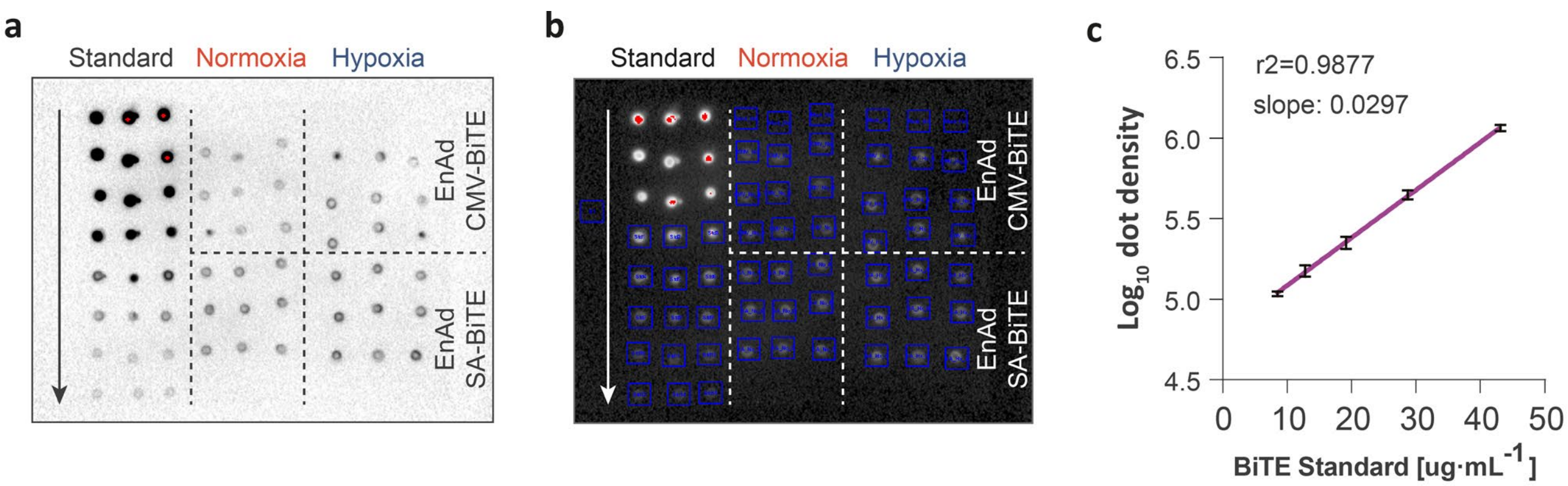

Supplementary Figure 6 EpCAM/CD3-BiTE secretion quantified via dot blot and densitometry. **a** The membrane shows serial dilutions of a purified EpCAM/CD3-bispecific T-cell engager (BiTE) standard, technical and biological replicates of experimental samples. **b, c** Samples were measured by densitometry to generate a standard curve and interpolate sample concentrations. Data are shown as mean ± SD.

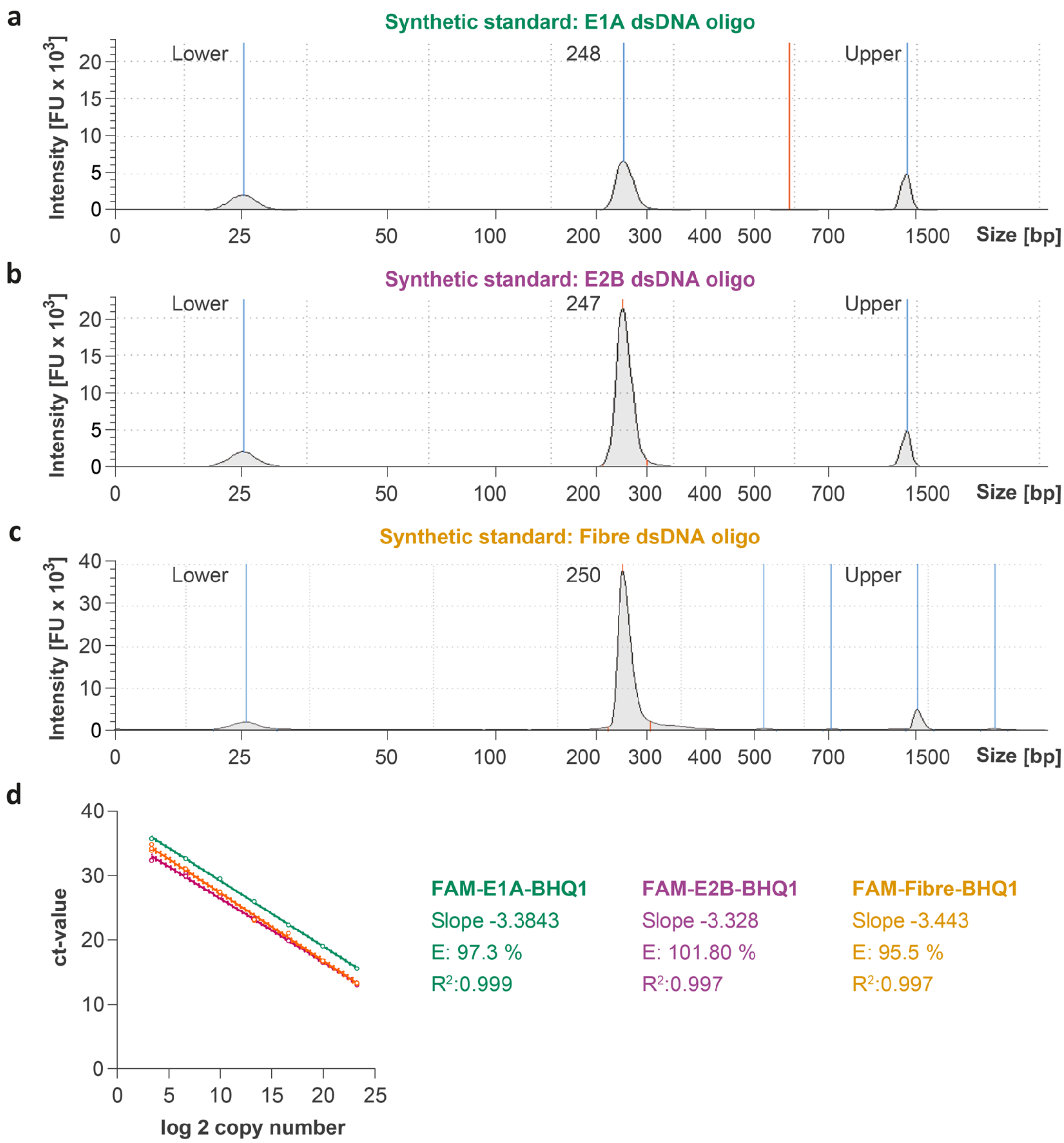

Supplementary Figure 7 Taqman assay for absolute quantification of viral RNA copy numbers. **a-c** Electropherograms of the synthetic DNA standards for absolute copy number quantification by qRT-PCR are shown. **d** PCR efficiency (E) of qPCR primer-probe sets, determined by linear regression using standards for fiber, E1A and E2B. Lines show the mean of interpolated values ± 95%CI.

Supplementary Tables

Supplementary Table 1 Adenovirus sequences considered for alignment (GenBank)

Index	Species	Genotype	Accession
1	A	Ad12	NC_001460
2	A	Ad18	GU191019
3	A	Ad31	AM749299
4	B	Ad3	DQ086466
5	B	Ad7	AC000018
6	B	Ad11p	AY598970
7	B	Ad14	AY803294
8	B	Ad16	AY601636
9	B	Ad21	AY601633
10	B	Ad34	AY737797
11	B	Ad35	AC000019
12	B	Ad50	AY737798
13	B	Ad55	KF908851
14	C	Ad1	AC000017
15	C	Ad2	AC000007
16	C	Ad5	AY339865
17	C	Ad6	LC068716
18	C	Ad57	HQ003817
19	E	Ad4	AY599837
20	F	Ad40	KU162869
21	F	Ad41	DQ315364
22	G	Ad52	DQ923122
23	D	Ad8	AB448769
24	D	Ad9	AJ854486
25	D	Ad10	JN226746
26	D	Ad13	JN226747
27	D	Ad15	AB562586
28	D	Ad17	AF108105
29	D	Ad19	AB448771
30	D	Ad20	JN226749
31	D	Ad22	FJ619037
32	D	Ad23	JN226750
33	D	Ad24	JN226751
34	D	Ad25	JN226752
35	D	Ad26	EF153474
36	D	Ad27	JN226753
37	D	Ad28	FJ824826
38	D	Ad29	JN226754
39	D	Ad30	JN226755
40	D	Ad32	JN226756
41	D	Ad33	JN226758
42	D	Ad36	GQ384080
43	D	Ad37	AB448778
44	D	Ad38	JN226759
45	D	Ad39	JN226760
46	D	Ad42	JN226761
47	D	Ad43	JN226762
48	D	Ad44	JN226763
49	D	Ad45	JN226764
50	D	Ad46	AY875648
51	D	Ad47	JN226757
52	D	Ad48	EF153473
53	D	Ad49	DQ393829
54	D	Ad51	JN226765
55	D	Ad53	AB605246
56	D	Ad54	NC012959
57	D	Ad56	HM770721
58	D	Ad58	HQ883276
59	D	Ad69	JN226748

Supplementary Table 2 Virus preparations

Batch	Virus	Promoter	Transgene/Tag	*vp $\cdot\mu g^{-1}$ viral DNA	Picogreen vp$\cdot mL^{-1}$	Plaque assay **PFU$\cdot mL^{-1}$
EJV-009	EnAd-SA-fLuc	Major late promoter	Firefly luciferase	2.83×10^{10}	1.670×10^{12}	5.35×10^{10}
EJV-010	EnAd-cFLAG	*E1A*	c-terminal FLAG	3.01×10^{10}	4.698×10^{11}	8.58×10^{9}
EJV-014	Ad5-E1A-fLuc	*E1A*	Firefly luciferase	2.59×10^{10}	1.459×10^{12}	8.77×10^{10}
EJV-017	EnAd-CMV-BiTE	*CMV*	EpCAM/CD3-BiTE	n.q.	n.q.	2.51×10^{10}
EJV-018	EnAd-SA-BiTE	Major late promoter	EpCAM/CD3-BiTE	n.q.	n.q.	3.16×10^{10}

* Constants derived from the genomic molecular weight to calculate virus particle (vp) titer from DNA Picogreen measurements, not quantified (n.q.). ** Plaque forming unit (pfu) measured by plaque assay in A549 cells. BiTE: Bi-specific T-cell engager.

Supplementary Table 3 Primers, probes and PCR conditions for Sybrgreen and Taqman assays

Primer	Gene	Locus	Concentration [nM]	Primer Sequence	Ta [°C]	PCR Efficiency [%]
Forward			400	CCATCTCCTGATTCTACTACC		
Reverse	*E1A*	Exon3	400	CCGTGTACTCAAGTCCAA	60	97.39
Probe			200	FAM-TAAGCCTGGGAAACGTCCAGCAGT-BHQ		
Forward			400	CTCTTCAATGATGTTACTTTCG		
Reverse	*E2B*	Exon1	400	GTAGCGAAGCGTGAGTAAG	60	101.80
Probe			200	FAM-AGGCTCCCTGTTCCCAGAGTTGGA-BHQ		
Forward			400	ACCGAAGAGCAATAAATG		
Reverse	*Fiber*	Exon1	400	TCGTCTTCTCTGATGTAG	60	95.5
Probe			200	FAM-TCGTATAACTTGGTCCTGGAACACA-BHQ		

* Ta: Annealing temperature

Supplementary Table 4 Antibodies and conditions for immunoblotting

Antibody target	Conjugate	Host	Dilution factor	*Block	Manufacturer	Cat Nr
Adenovirus	-	goat	20 000	Milk	Abcam	36851
Adenovirus 2/5 E1A	-	rabbit	1000	Milk	Santa Cruz	SC430
CAIX	-	mouse	1000	Milk	Bioscience Slovakia	M75
DAXX	-	rabbit	3000	BSA	CST	25C12
FLAG-tag	HRP	rat	20 000	Milk	Biolegend	637311
HIF-1α	-	mouse	1000	Milk	BD Biosciences	610958
HIF-2α		rabbit	1000	BSA	CST	7096/D9E3
Lamin A/C	-	mouse	1000	Milk	SantaCruz	7292
Puromycin	-	mouse	1000	Milk	Kerafast	EQ0001
α-Tubulin	-	mouse	2000	Milk	SantaCruz	5286
β-Actin	HRP	mouse	50 000	Milk	Sigma	A3854
anti-mouse	HRP	horse	2000	Milk	CST	7076
anti-rabbit	HRP	goat	2000	Milk	CST	7074
anti-goat	HRP	Donkey	20 000	Millk	Invitrogen	A16005

* 5% w/v in TBST (25mM Tris, 150 mM NaCl, 0.5% Tween-20, pH 7.6)

Supplementary Table 5 siRNAs used for RNA interference (Silencer Select siRNAs/ Thermo Fisher)

Target gene	Sense	Antisense
HIF1-a	UGUUUGUCAACCAACAGAGtt	UCAAGUUGCUGGUCAUCAGtt
HIF2-a	CAGCAUCUUUGAUAGCAGUtt	ACUGCUAUCAAAGAUGCUGtt
HIF1-β	GGCUCAAGGAGAUCGUUUAtt	UAAACGAUCUCCUUGAGCCca
DAXX-b	CUACAGAUCUCCAAUGAAAtt	UUUCAUUGGAGAUCUGUAGtg
DAXX-c	GGGAGUUGGAUCUCUCAGAAtt	UUCUGAGAGAUCCAACUCCtt

Supplementary Table 6 Primers and probes for ChIP quantification

Region	Forward primer	Reverse primer	Ta [°C]	PCR Efficiency [%]
E1A-HRE	GTATTTGTTCAGGGCCAGGTAG	CAGAAGACTTTGACACGGTACG	64.5	92.16
viral CDS	CAATCCCAACAGGCGTAACT	GACGTTGCAAGTTAGTGGGATA	60	92.55
NDRG1	TCCCTCCCAATCTCTCTCTTCTT	CACCATCAGCACAGCAAACTAC	65	93.58
CEBPB	CGTGCTTGGCTCTTTGTGAT	AGCCGCTTTTCAAATGAGCT	65	87.01

* Ta: Annealing temperature

Supplementary Table 7 Wash and elution buffers for ChIP

Low salt buffer

1	%	Triton
2	mM	EDTA
20	mM	Tris-HCl
150	%	NaCl
0.1	%	SDS

High salt buffer

1	%	Triton
2	mM	EDTA
20	mM	Tris-HCl
500	mM	NaCl
0.1	%	SDS

LiCl buffer

1	%	Igepal (NP40)
1	mM	EDTA (pH 8.0)
10	mM	Tris-HCl
250	mM	LiCl
1	%	SDS

TE buffer

10		Tris-HCl (pH 8.0)
1	mM	EDTA (pH 8.0)

Elution buffer

50	mM	Tris-HCl
10	mM	EDTA
1	%	SDS

ChIP dilution buffer

0.01	%	SDS
1.1	%	Triton-X100
1.2	mM	EDTA
16.7	mM	Tris-Hcl
167	mM	NaCl

Original Western blot images

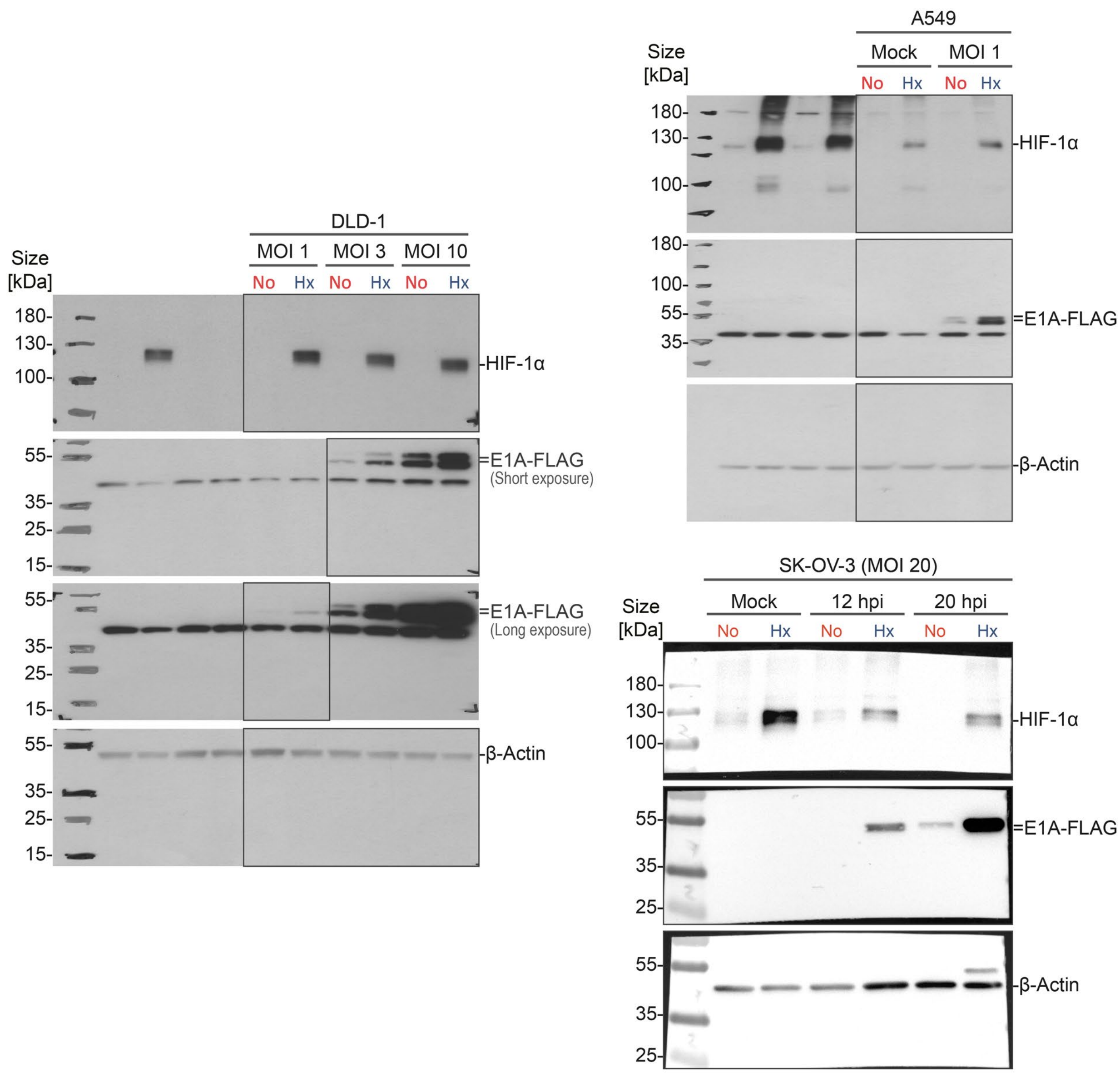

Original Western blot images to Figure 2b.

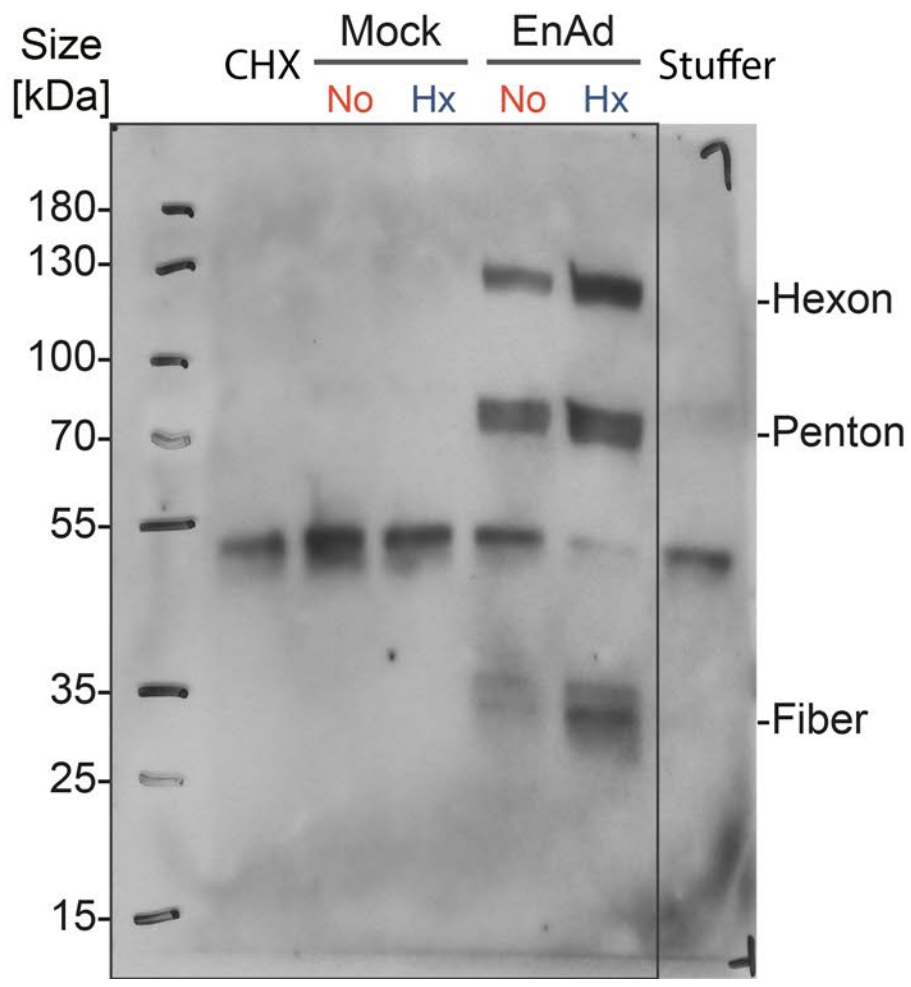

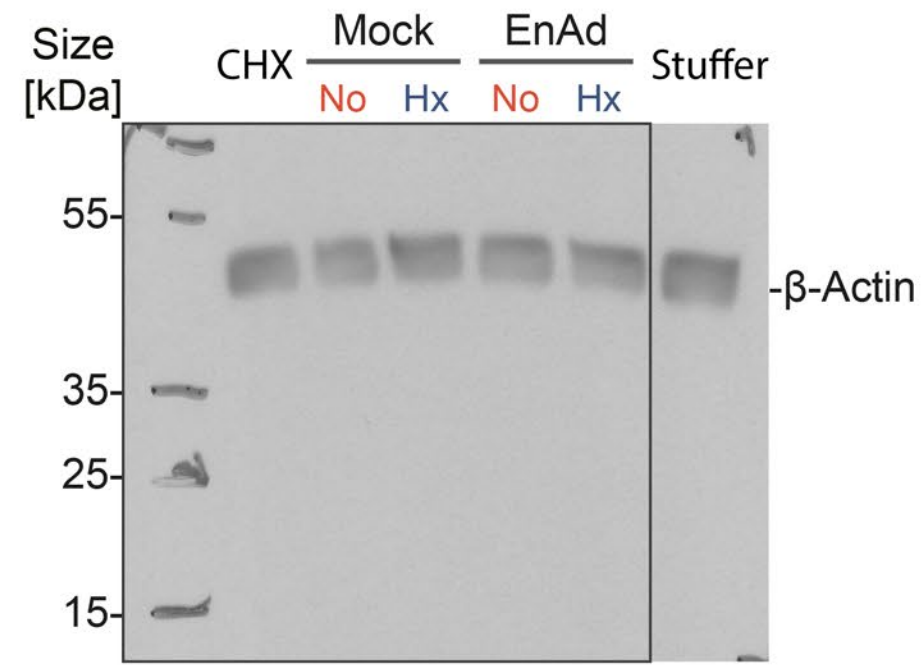

Original Western blot images to Figure 3b.

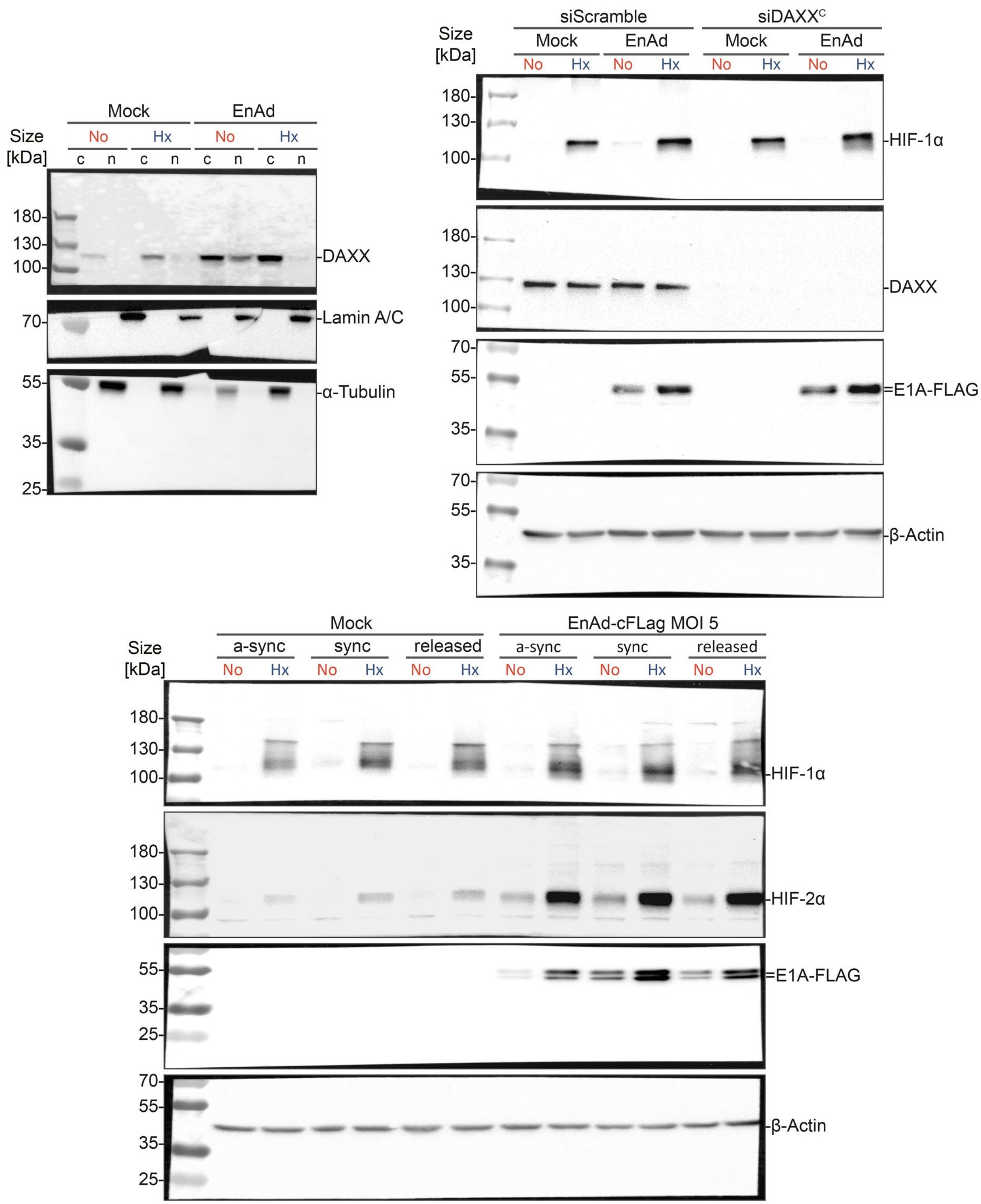

Original Western blot images to Figure 4.

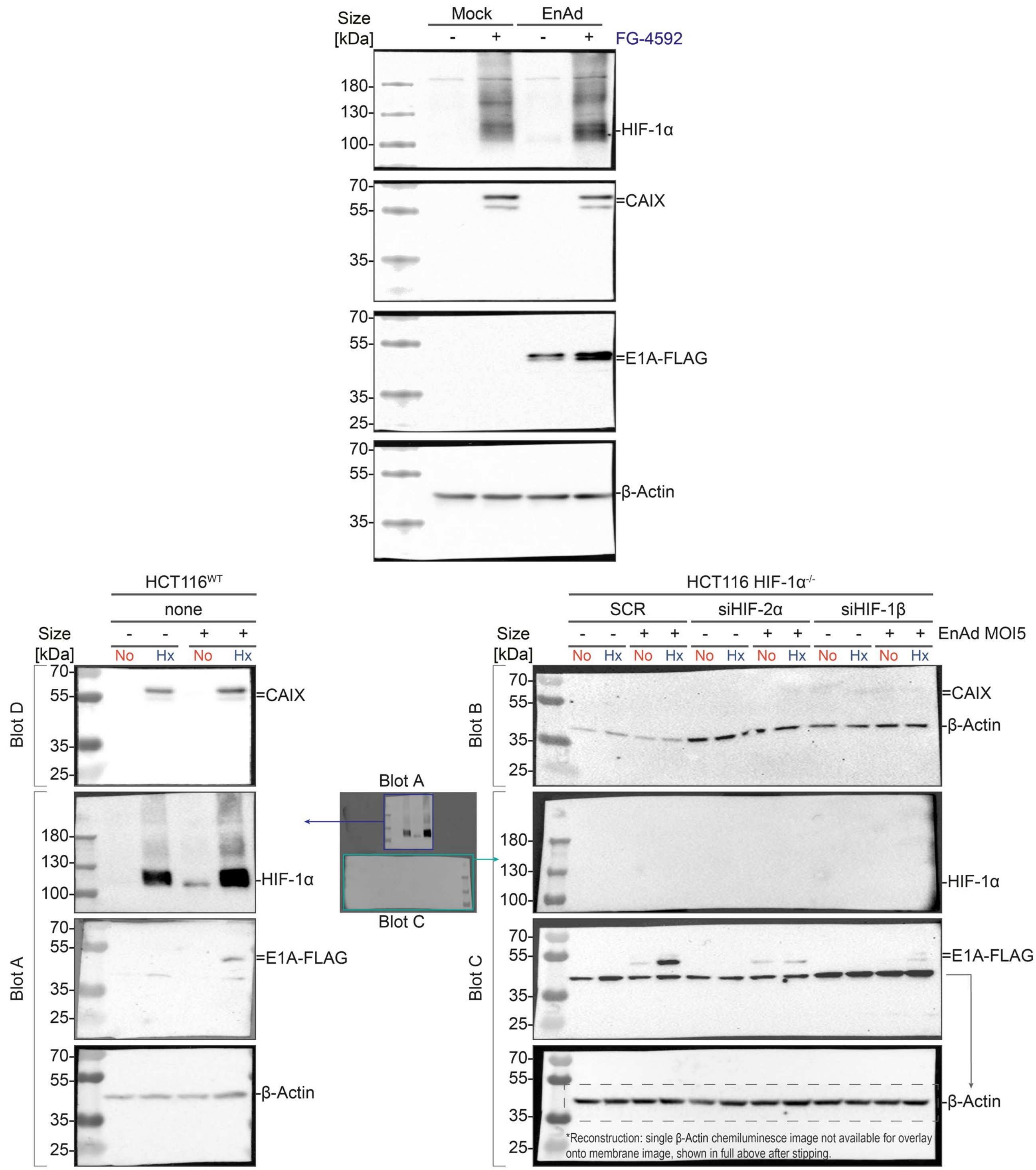

Original Western blot images to Figure 5.

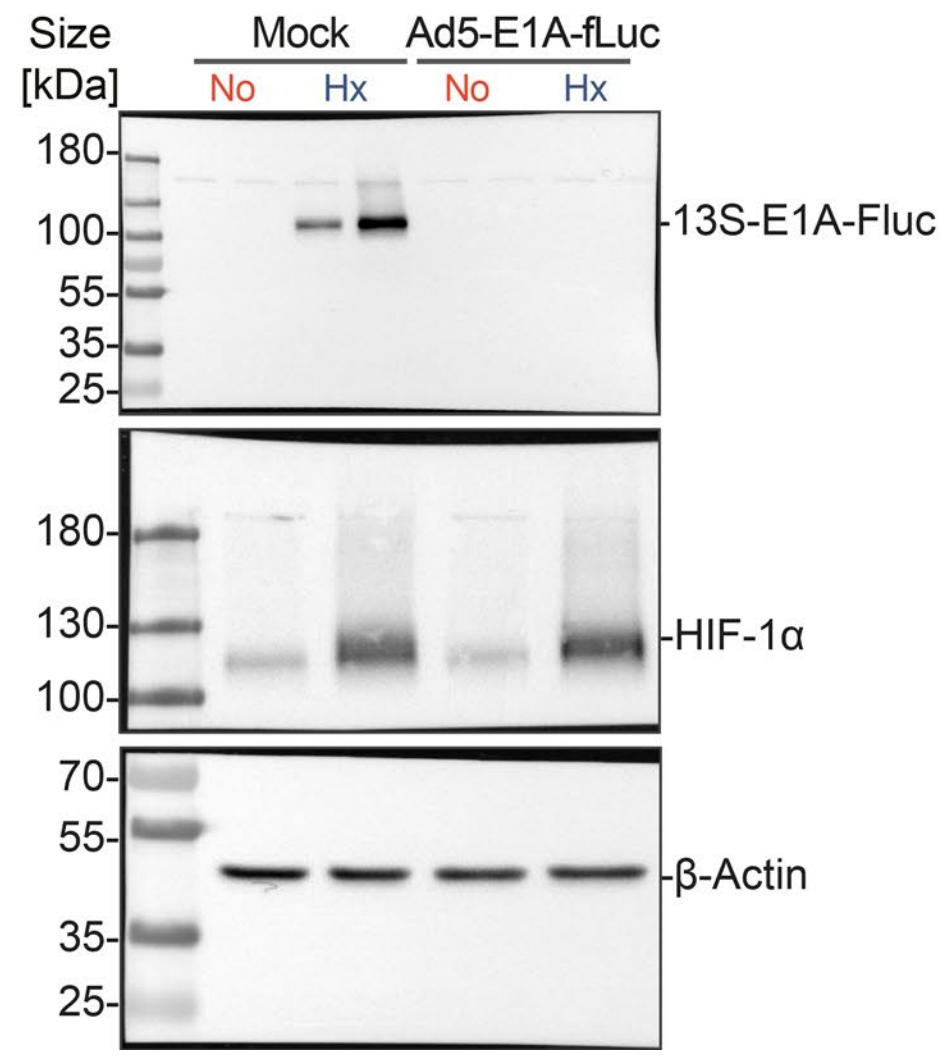

Original Western blot images to Supplementary Figure 5.
